# ﻿A checklist of the predators and parasitoids of the fall webworm *Hyphantriacunea* (Drury) (Lepidoptera, Erebidae) from around the world

**DOI:** 10.3897/zookeys.1211.123574

**Published:** 2024-09-09

**Authors:** Liang Ming Cao, Xiao Yi Wang, Toby R. Petrice, Therese M. Poland

**Affiliations:** 1 Key Laboratory of Forest Protection, State Forestry and Grassland Administration, Ecology and Nature Conservation Institute, Chinese Academy of Forestry, Beijing 100091, China Ecology and Nature Conservation Institute, Chinese Academy of Forestry Beijing China; 2 Northern Research Station, FS, USDA, 2601 Coolidge Rd, Ste 203, East Lansing, MI 48823, USA Northern Research Station, FS, USDA East Lansing United States of America

**Keywords:** China, distribution, Europe, floral differences, Natural enemy, North America

## Abstract

A checklist of 488 fall webworm *Hyphantriacunea* (Drury) natural enemies was compiled based on documentation in previous research across its world distribution, including 289 predators and 199 parasitoids. Predators in the checklist include 67 species from 17 families of Insecta, 1 species of Chilopoda, 183 species from 22 families of Arachnida, 1 species of Reptilia, 4 species from 2 families of Amphibia, 33 species from 18 families of Aves. In addition, the checklist includes fall webworm parasitoids from 18 families of Insecta. Among continents, 128 predators and 76 parasitoids were distributed in North America, 78 predators and 62 parasitoids in Asia, and 88 predators and 68 parasitoids in Europe.

## ﻿﻿Introduction

The fall webworm, *Hyphantriacunea* (Drury, 1773) (Lepidoptera: Erebidae), is native to North America, where it is considered to be a secondary pest (Tothill 1922). However, *H.cunea* has become a primary pest in some regions that it has invaded over the past 80 years (Edosa et al. 2019). During the 1940s, *H.cunea* invaded Hungary and Japan, and later spread to most European countries, all of East Asia, and parts of central Asia (Trajković and Žikić 2023). In 1979, *H.cunea* first invaded China where it has become a serious pest and its distribution has expanded annually (Ning et al. 2021).

Although *H.cunea* does not kill host trees in China, it causes aesthetic damage and is a nuisance pest, invading buildings, vehicles, and other structures during outbreaks (Ning et al. 2021). Also, cultural preferences in China do not tolerate the appearance of massive caterpillar nests in trees, which are much larger than those typically found in North America. It is unclear why *H.cunea* population levels are much higher in China compared to its native range; however, an important contributing factor may be a lack of natural enemies that have co-evolved with the pest in China. Therefore, it is necessary to compare the composition of *H.cunea* natural enemies in its native North American range to its invaded ranges in Europe and East Asia.

The research history on natural enemies of *H.cunea* can be divided into three stages: (1) prior to 1940 when *H.cunea* was only found in North America (Riley 1887a, b); (2) from 1940 to 1980, when *H.cunea* invaded Eastern Europe, Japan, and Korea, and natural enemy surveys were conducted in Europe, East Asia, and North America (Hasegawa and Itô 1967; Kayashima 1967; Warren and Tadić 1967; Tamura 1969; Kunimi 1983); and (3) from 1980 to present, after *H.cunea* invaded China and extensive surveys of natural enemies were conducted. During this most recent stage, numerous Chinese parasitoids and predators that attack *H.cunea* were documented (Yang et al. 2008, 2015a, b; Li 2011). More recently, surveys of natural enemies have also been conducted in some central Asian countries, such as Turkey and Iran (Sullivan et al. 2010, 2011, 2012; Karami et al. 2023). Our objective was to compile a global checklist of known natural enemies of *H.cunea*, assemble as much information as possible about these natural enemies, and ultimately build a database for selecting candidates for use in biological control of *H.cunea*. We reviewed the available *H.cunea* natural enemy literature across all time periods and assembled a summary of this information in a species checklist.

## ﻿﻿Materials and methods

### ﻿Natural enemy literature review

Our summary of *H.cunea* natural enemies is based on extensive literature searches through December 2023. A systematic literature search of CNKI (https://www.cnki.net/index/), Google Scholar (https://scholar.google.com/), Google (https://www.google.com), and Biodiversity Heritage Library (https://www.biodiversitylibrary.org/) was performed with key words, including “fall webworm”, “*Hyphantriacunea*”, “natural enemy”, “parasitoid”, “predator”. We included all literature related to predators and parasitoids of the fall webworm but did not include publications about pathogenic microorganisms of *H.cunea*. Overall, we reviewed 99 publications that reported information about natural enemies of *H.cunea*, from North America, Asia, and Europe.

### ﻿Natural enemy species information collected

For each publication, we gathered relevant information about the natural enemy species reported. We compiled information into a checklist of predator and parasitoid groups, organized by Latin family name. We also included the Chinese family name. Family names were validated by checking ITIS (Integrated Taxonomic Information System; ITIS 2023). Some species names reported in the earlier literature differed from current valid names. When this occurred, we transcribed the synonym after the citation (e.g., “Oliver 1963 as *Matiscarolina*”). For each species in this checklist, we summarized the following information: 1) distribution, 2) recorded interactions of predator or prey with *H.cunea*, 3) prey or host stage attacked, 4) parasitoid type, and 5) notes.

**Distribution.** The known geographic distribution of a given species, based on the most recent published literature or catalogue website. The level of detail about species distributions varied among publications, with some papers reporting country names (e.g., China, USA), geographical divisions or continents (e.g., North America, Asia), or zoogeographic divisions (e.g., Palearctic, Nearctic).

**Recorded interactions of predator or parasitoid with *H.cunea*.** The geographic distribution where each predator and parasitoid species was reported preying upon or parasitizing *H.cunea*. This information is very important for analyzing the geographical fauna of predators and parasitoids of the fall webworm and demonstrates the species biodiversity of natural enemies among the three major regions (North America, Europe, and East Asia).

**Prey stage.** The *H.cunea* developmental stage preyed upon (i.e., egg, larva, pupa, or adult).

**Host stage.** The developmental stage of *H.cunea* that is parasitized (i.e., egg, larva, or pupa).

**Parasitoid type.** Parasitoids were categorized based on location of host-feeding (ectoparasitoids or endoparasitoids); brood production (solitary or gregarious); whether they parasitize hosts directly or indirectly (primary or hyperparasitoids); host specificity (monophagous, oligophagous, or polyphagous); and their impact on their host (idiobiont parasitoids that paralyze their host and koinobiont parasitoids that do not).

**Notes.** Biological characters of each predator or parasitoid species including any additional information or clarification about the natural enemy species.

## ﻿﻿Checklist

### ﻿﻿Part I Predators

﻿**Insecta**

﻿**Mantodea**

﻿**Mantidae** 螳螂科

#### *Stagmomantiscarolina* (Johanson, 1763)

**Distribution.** Trinidad, Venezuela, Guatemala, Belize, Costa Rica, Mexico, Panama, USA (Soodnarinesingh 2015).

**Recorded interactions with *H.cunea*.** USA [Louisiana (Oliver 1963 as *Matiscarolina*)].

**Prey stage.** Larva (Oliver 1963).

**Notes.** Nymphs feed on small larva and adults feed on large larva (Oliver 1963).

#### *Tenoderasinensis* (Saussure, 1871)

**Distribution.** China, Japan, Micronesia, Thailand, North America, Canada (Patel and Singh 2016).

**Recorded interactions with *H.cunea*.** China [Beijing (Tao et al. 2008 as *Paratenoderaaridifolia*)].

##### ﻿﻿Orthoptera

﻿**Tettigoniidae** 螽斯科

#### *Tettigoniaviridissima* (Linnaeus, 1758)

**Distribution.** Europe and North Africa.

**Recorded interactions with *H.cunea*.** Europe (Warren and Tadić 1967).

**Prey stage.** Larva (Warren and Tadić 1967).

##### ﻿﻿Dermaptera

﻿**Forficulidae** 球螋科

#### *Forficulaauricularia* Linnaeus, 1758

**Distribution.** Europe, western Asia, North Africa, North America (Crumb et al. 1941).

**Recorded interactions with *H.cunea*.** Europe (Warren and Tadić 1967).

**Prey stage.** Egg (Warren and Tadić 1967).

##### ﻿﻿Hemiptera

﻿**Pentatomidae** 蝽科

#### *Apoecilusbracteatus* (Fitch, 1865)

**Distribution.** Canada, USA (Rider and Swanson 2021).

**Recorded interactions with *H.cunea*.** Canada [New Brunswick, Nova Scotia (Morris 1972)].

**Prey stage.** Larva (Morris 1972).

#### *Armacustos* (Fabricius, 1794)

**Distribution.** Europe, Asia (Zhao et al. 2018).

**Recorded interactions with *H.cunea*.** Europe (Warren and Tadić 1967), Italy (Boriani 1991). China [Shanghe County, Jinan City, Shandong Province (Wang et al. 2012 as *A.chinensis*)].

**Prey stage.** Egg (Boriani 1991), larva (Warren and Tadić 1967).

**Notes.** Found in 2.33–17.86% of *H.cunea* webs at surveyed sites in Liaoning Province (Wang et al. 2012).

#### *Euschistusservus* (Say, 1832)

**Distribution.** Canada, USA, Mexico (EPPO 2015).

**Recorded interactions with *H.cunea*.** USA (Riley 1887b).

**Prey stage.** Larva (Warren and Tadić 1967).

#### *Picromerusbidens* (Linnaeus, 1758)

**Distribution.** Europe, Asia (Rider 2006), North America: Canada, USA (Chordas 2015).

**Recorded interactions with *H.cunea*.** Europe (Warren and Tadić 1967).

**Prey stage.** Larva (Warren and Tadić 1967).

#### *Pinthaeussanguinipes* (Fabricius, 1781)

**Distribution.** Europe, Asia (Zhao et al. 2013).

**Recorded interactions with *H.cunea*.** Europe (Warren and Tadić 1967).

**Prey stage.** Larva (Warren and Tadić 1967).

#### *Podisusmaculiventris* (Say, 1832)

**Distribution.** Mexico, Bahamas, USA, Canada (De Clercq 2008).

**Recorded interactions with *H.cunea*.** USA [Arkansas (Tadić 1963)], Warren and Tadić (1967) also listed as *P.modestus*, a synonym of *P.maculiventris* (Phillips 1983)].

**Prey stage.** Larva (Warren and Tadić 1967).

#### *Podisusplacidus* Uhler, 1870

**Distribution.** USA, Canada (Phillips 1983).

**Recorded interactions with *H.cunea*.** USA [Arkansas (Tadić 1963)].

**Prey stage.** Larva (Warren and Tadić 1967).

#### *Podisusserieventris* Uhler, 1871

**Distribution.** USA, Canada (Phillips 1983).

**Recorded interactions with *H.cunea*.** USA (Warren and Tadić 1967).

**Prey stage.** Larva (Warren and Tadić 1967).

##### ﻿﻿Anthocoridae 花蝽科

#### *Oriusmajusculus* (Reuter, 1879)

**Distribution.** Europe, Canada (Henry 2008).

**Recorded interactions with *H.cunea*.** Europe (Warren and Tadić 1967).

**Prey stage.** Egg (Warren and Tadić 1967).

##### ﻿﻿Nabidae 姬蝽科

#### *Himacerusapterus* (Fabricius, 1798)

**Distribution.** China, Europe, Canada [Nova Scotia (Lartvière 1992)].

**Recorded interactions with *H.cunea*.** Europe (Warren and Tadić 1967 as *Nabisapterus*), China [Dandong City, Liaoning Province (Shu and Yu 1985)].

**Prey stage.** Egg (Shu and Yu 1985), larva (Warren and Tadić 1967; Shu and Yu 1985).

**Notes.** Both nymph and adult consume *H.cunea* larvae (Shu and Yu 1985).

##### ﻿﻿Reduviidae 猎蝽科

#### *Agriosphodrusdohrni* (Signoret, 1862)

**Distribution.** China, Japan, India, Vietnam.

**Recorded interactions with *H.cunea*.** China [Beijing, laboratory feeding and testing in 2019].

**Prey stage.** Larva.

**Notes.** Both nymph and adult consume *H.cunea* larvae in the laboratory; mature nymphs can consume 4–7 larvae per day, adults can consume 4–11 larvae per day (unpublished data, LMC).

#### *Ariluscristatus* (Linnaeus, 1763)

**Distribution.** Canada, USA, Mexico, Guatemala (Blatchley 1926).

**Recorded interactions with *H.cunea*.** USA [Arkansas (Tadić 1963), Louisiana (Oliver 1963, 1964)].

**Prey stage.** Larva (Tadić 1963).

**Notes.** Both nymph and adult consume larvae of *H.cunea* (Tadić 1963; Oliver 1963), attacks second through seventh larval instars (Oliver 1964).

#### *Pselliopuscinctus* (Fabricius, 1776)

**Distribution.** Canada, USA.

**Recorded interactions with *H.cunea*.** USA [Arkansas (Tadić 1963), Louisiana (Oliver 1964)].

**Prey stage.** Larva (Oliver 1964).

**Notes.** Attacks second through fifth larval instars (Oliver 1964).

#### *Rhynocorisiracundus* (Poda, 1761)

**Distribution.** Europe, West and Middle Asia (Putshkov and Putshkov 1996).

**Recorded interactions with *H.cunea*.** Europe (Warren and Tadić 1967 as *Harpactoriracundus*)

**Prey stage.** Larva (Warren and Tadić 1967).

#### *Sineaspinipes* (Herrich-Schäffer, 1846)

**Distribution.** USA (from New York south to Florida and west to South Dakota, Colorado, and Texas), Mexico (Froeschner 1988).

**Recorded interactions with *H.cunea*.** USA [Arkansas (Tadić 1963), Louisiana (Oliver 1964)].

**Prey stage.** Larva (Oliver 1964).

**Notes.** Attacks first through fourth larval instars (Oliver 1964).

#### *Stenopodacinerea* Laporte, 1833

**Distribution.** Canada, USA, Mexico (Froeschner 1988), Argentina (Diez and Coscarón 2014).

**Recorded interactions with *H.cunea*.** USA [Arkansas (Tadić 1963)].

**Prey stage.** Larva (Tadić 1963).

#### *Yolinusalbopustulatus* China, 1940

**Distribution.** China, Japan, Vietnam (Lam et al. 2015).

**Recorded interactions with *H.cunea*.** China [Beijing, laboratory feeding and testing in 2019].

**Prey stage.** Larva.

**Notes.** Attacks *H.cunea* in the laboratory, one adult can consume 8–13 larvae per day (unpublished data, LMC).

#### *Zeluslongipes* (Linnaeus, 1767)

**Distribution.** Southern parts of USA, Mexico, Central America, the Caribbean, northern South America, Paraguay, and southern Brazil (Zhang et al. 2016).

**Recorded interactions with *H.cunea*.** USA [Melville (Oliver 1963 as *Z.bilobus*)].

**Prey stage.** Larva (Oliver 1963).

**Notes.** Attacks fourth through sixth larval instars (Oliver 1964).

#### *Zeluscervicalis* Stål, 1872

**Distribution.** Belize, Colombia, Costa Rica, El Salvador, Guatemala, Honduras, Mexico, USA (Zhang et al. 2016).

**Recorded interactions with *H.cunea*.** USA [Lacombe (Oliver 1963)].

**Prey stage.** Larva (Oliver 1963).

**Notes.** Attacks fourth through sixth larval instars (Oliver 1964).

#### *Zelusluridus* Stål, 1862

**Distribution.** Canada, Mexico, and USA (Zhang et al. 2016).

**Recorded interactions with *H.cunea*.** USA [Arkansas (Tadić 1963 as *Z.exsanguis*)].

**Prey stage.** Larva (Tadić 1963 as *Z.exsanguis*).

**Notes.** Almost all specimens collected from the US were misidentified as *Z.exsanguis* (Zhang et al. 2016).

#### *Zelustetracanthus* Stål, 1862

**Distribution.** Brazil, Costa Rica, Curaçao, Honduras, Mexico, Panama, Paraguay, USA and Venezuela (Zhang et al. 2016).

**Recorded interactions with *H.cunea*.** USA [Arkansas (Tadić 1963 as *Z.socius*)].

**Prey stage.** Larva (Tadić 1963 as *Z.exsanguis*).

##### ﻿﻿Miridae 盲蝽科

#### *Deraeocorisruber* (Linnaeus, 1758)

**Distribution.** Britain, Corsica, Denmark, France, Germany, Italy, Macedonia, Moravia, Spain, Sweden, USA, Canada (Schuh 2016).

**Recorded interactions with *H.cunea*.** Europe (Warren and Tadić 1967).

**Prey stage.** Egg (Warren and Tadić 1967).

##### ﻿﻿Neuroptera

﻿**Chrysopidae** 草蛉科

#### *Chrysopacarnea* Stephens, 1836

**Distribution.** Palaearctic, Afrotropical, Oriental (Oswald 2007).

**Recorded interactions with *H.cunea*.** Italy (Boriani 1991), Turkey [Düzce (Avci et al. 2022)].

**Prey stage.** Egg (Boriani 1991).

#### *Chrysopaformosa* Brauer, 1851

**Distribution.** Palaearctic, widespread (Oswald 2007).

**Recorded interactions with *H.cunea*.** China [Dandong city, Liaoning province (Shu and Yu 1985)].

**Prey stage.** Egg and larva (Shu and Yu 1985).

#### *Chrysopaoculata* Say, 1839

**Distribution.** Canada, USA, Mexico (Oswald 2007).

**Recorded interactions with *H.cunea*.** North America (Warren and Tadić 1967).

**Prey stage.** Egg and larva (Warren and Tadić 1967).

#### *Chrysopaperla* (Linnaeus, 1758)

**Distribution.** Palaearctic, widespread (Oswald 2007).

**Recorded interactions with *H.cunea*.** Europe (Warren and Tadić 1967).

**Prey stage.** Egg and larva (Warren and Tadić 1967).

#### *Chrysopaquadripunctata* Burmeister, 1839

**Distribution.** Southern Canada, eastern USA, Mexico (Oswald 2007).

**Recorded interactions with *H.cunea*.** USA [Arkansas (Tadić 1963)].

**Prey stage.** Egg and larva (Warren and Tadić 1967).

#### *Chrysopapallens* (Rambur, 1838)

**Distribution.** Palaearctic, widespread (Oswald 2007).

**Recorded interactions with *H.cunea*.** China [Dandong City, Liaoning Province (Shu and Yu 1985 as *C.septempunctata*), Liaocheng City, Shandong Province (Yue et al. 2016 as *C.septempunctata*)].

**Prey stage.** Egg (Shu and Yu 1985), larva (Yue et al. 2016).

#### *Chrysopanipponensis* (Okamoto, 1914)

**Distribution.** China, Japan, Korea, Mongolia, Philippines, eastern Russia (Oswald 2007).

**Recorded interactions with *H.cunea*.** China [Liaocheng City, Shandong Province (Yue et al. 2016 as *C.sinica*)].

**Prey stage.** Larva (Yue et al. 2016).

#### *Chrysopacarnea* (Stephens, 1836)

**Distribution.** Palaearctic (widespread), Afrotropical (Cape Verde, Oman, United Arab Emirates, Yemen), Oriental (China, India, Nepal) (Oswald 2007).

**Recorded interactions with *H.cunea*.** Europe (Warren and Tadić 1967 as *C.vulgaris*).

**Prey stage.** Egg and larva (Warren and Tadić 1967).

##### ﻿﻿Panorpidae 蝎蛉科

#### *Panorpacommunis* Linnaeus, 1758

**Distribution.** Asia, Europe (Penny and Byers 1979).

**Recorded interactions with *H.cunea*.** Europe (Warren and Tadić 1967).

**Prey stage.** Egg (Warren and Tadić 1967).

##### ﻿﻿Coleoptera

﻿**Cantharidae** 花萤科

#### *Cantharisfusca* Linnaeus, 1758

**Distribution.** Western and central Europe (Kazantsev 2011).

**Recorded interactions with *H.cunea*.** Europe (Warren and Tadić 1967).

**Prey stage.** Larva (Warren and Tadić 1967).

#### *Cantharisrufa* (Linnaeus, 1758)

**Distribution.** Europe, Canada (Pelletier and Hébert 2014).

**Recorded interactions with *H.cunea*.** Europe (Warren and Tadić 1967).

**Prey stage.** Larva (Warren and Tadić 1967).

##### ﻿﻿Carabidae 步甲科

#### *Calosomainquisitor* (Linnaeus, 1758)

**Distribution.** Europe and part of the Mediterranean (Bruschi 2010).

**Recorded interactions with *H.cunea*.** Europe (Warren and Tadić 1967).

**Prey stage.** Egg (Warren and Tadić 1967).

#### *Calosomascrutator* (Fabricius, 1775)

**Distribution.** Canada, El Salvador, Guatemala, Honduras, Mexico, Nicaragua, Puerto Rico, USA, Venezuela (Bruschi 2010).

**Recorded interactions with *H.cunea*.** North America (Warren and Tadić 1967).

**Prey stage.** Larva and adult (Warren and Tadić 1967).

#### *Calosomasycophanta* (Linnaeus, 1758)

**Distribution.** Northern Africa and throughout Europe (Bruschi 2010).

**Recorded interactions with *H.cunea*.** Europe (Warren and Tadić 1967).

**Prey stage.** Egg and larva (Warren and Tadić 1967).

#### *Carabushortensis* Linnaeus, 1758

**Distribution.** Europe (Turin et al. 2003).

**Recorded interactions with *H.cunea*.** Europe (Warren and Tadić 1967).

**Prey stage.** Larva (Warren and Tadić 1967).

#### *Chlaeniuspallipes* (Gebler, 1823)

**Distribution.** China, North Korea, South Korea, Japan, Russia, Mongolia (Lorenz 2021).

**Recorded interactions with *H.cunea*.** China [Dandong City, Liaoning Province (Shu and Yu 1985)].

**Prey stage.** Pupa (Shu and Yu 1985).

#### *Nebrialivida* (Linnaeus, 1758)

**Distribution.** China, Japan, Russa, Europe (Liang and Yu 2000).

**Recorded interactions with *H.cunea*.** China [Liaocheng City, Shandong Province (Yue et al. 2016)].

**Prey stage.** Larva (Yue et al. 2016).

#### *Parenacavipennis* (Bates, 1873)

**Distribution.** China, Japan, North Korea, South Korea, Vietnam, Nepal(?) (Shi and Liang 2023).

**Recorded interactions with *H.cunea*.** China [Rongcheng City, Shandong Province (Wang et al. 1999)].

**Prey stage.** Larva (Wang et al. 1999).

**Notes.** Both adults and larvae prey on *H.cunea* larvae. Adults prey at night on all instars; *P.cavipennis* larvae prey on caterpillars in webs (Wang et al. 1999).

#### *Parenalaesipennis* (Bates, 1873)

**Distribution.** China, Japan, South Korea, Vietnam, Laos, Thailand, Myanmar, Malaysia, India, Nepal (Shi and Liang 2023).

**Recorded interactions with *H.cunea*.** China [Dalian City, Liaoning Province (Yang et al. 2008)].

**Prey stage.** Larva (Yang et al. 2008).

**Notes.** Both adults and larvae prey on *H.cunea* and live inside the host web; consume 110–265 larvae per web; consume all larvae in 3.2% of webs in the Dalian area (Yang et al. 2008).

#### *Parenalatecincta* (Bates, 1873)

**Distribution.** Japan, South Korea, Russia, China, Vietnam, Laos, Thailand, India, Nepal, the Philippines (Shi and Liang 2023).

**Recorded interactions with *H.cunea*.** China [Dalian City, Liaoning Province (Yang et al. 2008)].

**Prey stage.** Larva (Yang et al. 2008).

**Notes.** Similar biology to *P.laesipennis* (Yang et al. 2008).

#### *Plochionustimidus* Haldeman, 1843

**Distribution.** Canada, Mexico, USA (Bousquet 2012).

**Recorded interactions with *H.cunea*.** USA [Arkansas (Tadić 1963)].

**Prey stage.** Larva (Tadić 1963).

**Notes.** Both adult and larva prey on *H.Cunea* larvae. Adult tears larva into pieces and then consumes each piece except for the head capsule (Tadić 1963).

##### ﻿﻿Coccinellidae 瓢虫科

#### *Coccinellaseptempunctata* Linnaeus, 1758

**Distribution.** Native to temperate Europe, North Africa, and Asia, established in North America and South America [Brazil, Chile] (Beverley 2022).

**Recorded interactions with *H.cunea*.** China [Dandong City, Liaoning Province (Shu and Yu 1985), Liaocheng City, Shandong Province (Yue et al. 2016)].

**Prey stage.** Egg (Yue et al. 2016).

**Notes.** Larva of *C.septempunctata* prey on eggs (Yue et al. 2016).

#### *Harmoniaaxyridis* (Pallas, 1773)

**Distribution.** Native to central and eastern Asia, introduced to Europe, North America, South America, the Middle East, South Africa, and Australia (Roy 2022).

**Recorded interactions with *H.cunea*.** China [Beijing (unpublished data, LMC), Liaocheng City, Shandong Province (Yue et al. 2016)].

**Prey stage.** Egg (Yue et al. 2016).

**Notes.** Larva of *H.axyridis* prey on eggs (Yue et al. 2016).

#### *Propyleaquatuordecimpunctata* (Linnaeus, 1758)

**Distribution.** Europe, North Africa, the Russian Far East, Asia, North America (Nikitsky and Ukrainsky 2016).

**Recorded interactions with *H.cunea*.** Europe (Warren and Tadić 1967).

**Prey stage.** Larva (Warren and Tadić 1967).

##### ﻿﻿Silphidae 葬甲科

#### *Dendroxenaquadrimaculata* (Scopoli, 1771)

**Distribution.** Central and Southern Europe, Turkey, Iran and Kazakhstan, Northern Africa, Northern America (Stolbov and Sergeeva 2020).

**Recorded interactions with *H.cunea*.** Europe (Warren and Tadić 1967 as *Xylodrepapunctata*).

**Prey stage.** Egg and larva (Warren and Tadić 1967).

#### *Silphacarinata* Herbst, 1783

**Distribution.** From Europe to Transbaikalia and Central Asia, including Mongolia and westernmost China (Nishikawa et al. 2010).

**Recorded interactions with *H.cunea*.** Europe (Warren and Tadić 1967).

**Prey stage.** Egg and larva (Warren and Tadić 1967).

##### ﻿﻿Staphylinidae 隐翅虫科

#### *Quediusochripennis* (Ménétriés, 1832)

**Distribution.** West Palaearctic, Mediterranean, North Africa, Oriental (Salnitska and Solodovnikov 2019).

**Recorded interactions with *H.cunea*.** Europe (Warren and Tadić 1967).

**Prey stage.** Egg and larva (Warren and Tadić 1967).

##### ﻿﻿Hymenoptera

﻿**Formicidae** 蚁科

#### *Formicarufa* Linnaeus, 1761

**Distribution.** Nearctic, Palaearctic (AntWeb, 2023).

**Recorded interactions with *H.cunea*.** Europe (Warren and Tadić 1967).

**Prey stage.** Larva (Warren and Tadić 1967).

#### *Linepithemahumile* (Mayr, 1868)

**Distribution.** Afrotropical, Australasia, Indomalaya, Nearctic, Neotropical, Oceania, Palaearctic (AntWeb 2023).

**Recorded interactions with *H.cunea*.** Europe (Warren and Tadić 1967 as *Iridomyrmexhumilis*).

**Prey stage.** Larva (Warren and Tadić 1967).

#### *Solenopsissaevissima* (Smith, 1855)

**Distribution.** Afrotropical, Neotropical (AntWeb 2023).

**Recorded interactions with *H.cunea*.** Europe (Warren and Tadić 1967).

**Prey stage.** Larva (Warren and Tadić 1967).

##### ﻿﻿Vespidae 胡蜂科

#### *Dolichovespulaarenaria* (Fabricius, 1775)

**Distribution.** Abundant throughout boreal North America (Kimsey and Carpenter 2012).

**Recorded interactions with *H.cunea*.** Canada [New Brunswick (Morris 1972 as *Vespulaarenaria*)].

**Prey stage.** Larva (Morris 1972).

#### *Dolichovespulamaculate* (Linnaeus, 1763)

**Distribution.** North America (Kimsey and Carpenter 2012).

**Recorded interactions with *H.cunea*.** Canada [New Brunswick (Morris 1972 as *Vespulamaculata*)].

**Prey stage.** Larva (Morris 1972).

#### *Dolichovespulanorvegicoides* (Sladen, 1918)

**Distribution.** Widely throughout northern North America and further south along mountain ranges (Kimsey and Carpenter 2012).

**Recorded interactions with *H.cunea*.** Canada [New Brunswick (Morris 1972 as *Vespulanorvegicoides*)].

**Prey stage.** Larva (Morris 1972).

#### *Polistesannularis* (Linnaeus, 1763)

**Distribution.** USA (Carpenter 1996).

**Recorded interactions with *H.cunea*.** North America (Warren and Tadić 1967).

**Prey stage.** Larva and adult (Warren and Tadić 1967).

#### *Polistesdominulus* (Christ, 1791)

**Distribution.** Central and southern Europe, Turkey, northern Africa, Israel, Syria, Afghanistan, Russia, Iran, Uzbekistan, Turkmenistan, Pakistan, India, Mongolia, China; introduced to Australia, Chile, USA (Carpenter 1996).

**Recorded interactions with *H.cunea*.** Europe (Warren and Tadić 1967 as *P.dominula*).

**Prey stage.** Larva (Warren and Tadić 1967).

#### *Polistesarizonensis* Snelling, 1954

**Distribution.** USA (Carpenter 1996).

**Recorded interactions with *H.cunea*.** North America (Warren and Tadić 1967 as *P.exclamans*).

**Prey stage.** Larva and adult (Warren and Tadić 1967).

#### *Polistesaurifer* Saussure, 1853

**Distribution.** Canada, Mexico, USA (Carpenter 1996).

**Recorded interactions with *H.cunea*.** North America (Warren and Tadić 1967 as *P.fuscatus*).

**Prey stage.** Larva and adult (Warren and Tadić 1967).

#### *Polistesmetricus* Say, 1831

**Distribution.** USA (Carpenter 1996).

**Recorded interactions with *H.cunea*.** North America (Warren and Tadić 1967).

**Prey stage.** Larva and adult (Warren and Tadić 1967).

#### *Polistesfuscatus* (Fabricius, 1793)

**Distribution.** Canada, USA (Carpenter 1996).

**Recorded interactions with *H.cunea*.** North America (Warren and Tadić 1967 as *P.pallipes*).

**Prey stage.** Larva (Warren and Tadić 1967).

#### *Vespulaalascensis* (Packard, 1870)

**Distribution.** North America (Kimsey and Carpenter 2012).

**Recorded interactions with *H.cunea*.** Canada [New Brunswick (Morris 1972 as *V.vulgaris*)], USA [Roycefield (Smulyan 1924 as *Vespacommunis*)].

**Prey stage.** Larva (Smulyan 1924; Morris 1972).

**Notes.** Preys on larvae by piercing the integument with their mandibles and consuming the liquid and softer parts (Smulyan 1924).

#### *Vespulamaculifrons* (du Buysson, 1905)

**Distribution.** Canada, USA (Kimsey and Carpenter 2012).

**Recorded interactions with *H.cunea*.** Canada [New Brunswick (Morris 1972 as *V.maculata*)].

**Prey stage.** Larva (Morris 1972).

##### ﻿﻿Chilopoda

﻿**Scutigeromorpha**

﻿**Scutigeridae** 蚰蜒科

#### *Thereuopodaclunifera* Wood, 1862

**Distribution.** China, Japan (Würmli 1979).

**Recorded interactions with *H.cunea*.** Japan (Hasegawa and Itô 1967).

**Prey stage.** Adult (Hasegawa and Itô 1967).

**Notes.** Occasionally preys on *H.cunea* (Hasegawa and Itô 1967).

##### ﻿﻿Arachnida

﻿**Araneae**

﻿**Agelenidae** 漏斗蛛科

#### *Agelenalimbata* Thorell, 1897

**Distribution.** China, Myanmar, Laos (World Spider Catalog 2023), Japan (Kunimi 1983).

**Recorded interactions with *H.cunea*.** Japan [Fuchu (Kunimi 1983)].

**Prey stage.** Larva (Kunimi 1983).

**Notes.** Collected from fall webworm nest (Kunimi 1983).

#### *Agelenopsis* sp.

**Recorded interactions with *H.cunea*.** USA [Arkansas (Warren et al. 1967)].

**Prey stage.** Larva (Warren et al. 1967).

**Notes.** Collected from fall webworm nest (Warren et al. 1967).

#### *Allagelenadifficilis* (Fox, 1936)

**Distribution.** China, Korea (World Spider Catalog 2023).

**Recorded interactions with *H.cunea*.** China [Dandong City, Liaoning Province (Shu and Yu 1985 as *Agelenadifficilis*)].

**Prey stage.** Larva (Shu and Yu 1985).

#### *Allagelenaopulenta* (L. Koch, 1878)

**Distribution.** Russia (Far East), Korea, Japan (World Spider Catalog 2023).

**Recorded interactions with *H.cunea*.** Japan [Fuchu (Kunimi 1983 as *Agalenaopulenta*)].

**Prey stage.** Larva (Kayashima 1967; Kunimi 1983).

**Notes.** Collected from fall webworm nest (Kunimi 1983).

##### ﻿﻿Anyphaenidae 近管蛛科

#### *Anyphaenaceler* (Hentz, 1847)

**Distribution.** Canada, USA (World Spider Catalog 2023).

**Recorded interactions with *H.cunea*.** USA [Arkansas (Warren et al. 1967)].

**Prey stage.** Larva (Warren et al. 1967).

**Notes.** Collected from fall webworm nest (Warren et al. 1967).

#### *Anyphaenamaculata* (Banks, 1896)

**Distribution.** USA (World Spider Catalog 2023).

**Recorded interactions with *H.cunea*.** USA [Arkansas (Warren et al. 1967)].

**Prey stage.** Larva (Warren et al. 1967).

**Notes.** Collected from fall webworm nest (Warren et al. 1967).

#### *Anyphaena* sp.

**Recorded interactions with *H.cunea*.** USA [Arkansas (Warren et al. 1967)].

**Prey stage.** Larva (Warren et al. 1967).

**Notes.** Collected from fall webworm nest (Warren et al. 1967).

#### *Aysha* sp.

**Recorded interactions with *H.cunea*.** USA [Arkansas (Warren et al. 1967)].

**Prey stage.** Larva (Warren et al. 1967).

**Notes.** Collected from fall webworm nest (Warren et al. 1967).

#### *Hibanagracilis* (Hentz, 1847)

**Distribution.** Canada, USA, Jamaica (World Spider Catalog 2023).

**Recorded interactions with *H.cunea*.** USA [Arkansas (Warren et al. 1967 as *Ayshagracilis*)].

**Prey stage.** Larva (Warren et al. 1967).

**Notes.** Collected from fall webworm nest (Warren et al. 1967).

#### *Lupettianamordax* (O. Pickard-Cambridge, 1896)

**Distribution.** USA to Peru, Brazil (World Spider Catalog 2023).

**Recorded interactions with *H.cunea*.** USA [Arkansas (Warren et al. 1967 as *Anyphaenafragilis*)].

**Prey stage.** Larva (Warren et al. 1967).

**Notes.** Collected from fall webworm nest (Warren et al. 1967).

#### *Wulfilasaltabundus* (Hentz, 1847)

**Distribution.** USA, Canada (World Spider Catalog 2023).

**Recorded interactions with *H.cunea*.** USA [Arkansas (Warren et al. 1967 as *Anyphaenellasaltabunda*)].

**Prey stage.** Larva (Warren et al. 1967).

**Notes.** Collected from fall webworm nest (Warren et al. 1967).

##### ﻿﻿Araneidae 圆蛛科

#### *Araneusdiadematus* Clerck, 1757

**Distribution.** Europe, Middle East, Turkey, Caucasus, Russia (Europe to Far East), Iran, Central Asia, China, Japan. Introduced to North America (World Spider Catalog 2023).

**Recorded interactions with *H.cunea*.** South of the European part of former USSR (Sharov et al. 1984), Italy [Po Valley (Groppali et al. 1993), Pavia (Camerini and Groppali 1999)].

**Prey stage.** Larva (Groppali et al. 1993; Camerini and Groppali 1999).

#### *Aranielladisplicata* (Hentz, 1847)

**Distribution.** North America, Europe, Russia (Europe to Far East), Kazakhstan, China, Korea, Japan (World Spider Catalog 2023).

**Recorded interactions with *H.cunea*.** USA [Arkansas (Warren et al. 1967)].

**Prey stage.** Larva (Warren et al. 1967).

**Notes.** Collected from fall webworm nest (Warren et al. 1967).

#### *Araneuscingulatus* (Walckenaer, 1841)

**Distribution.** USA (World Spider Catalog 2023).

**Recorded interactions with *H.cunea*.** USA [Arkansas (Warren et al. 1967 as *Conepeiraozarkensis*)].

**Prey stage.** Larva (Warren et al. 1967).

**Notes.** Collected from fall webworm nest (Warren et al. 1967).

#### *Araneuscirce* (Audouin, 1826)

**Distribution.** Southern Europe, Egypt, Turkey, Caucasus, Iran (World Spider Catalog 2023).

**Recorded interactions with *H.cunea*.** USA [Arkansas (Warren et al. 1967 as *Epeiracornuta*)].

**Prey stage.** Larva (Warren et al. 1967).

**Notes.** Collected from fall webworm nest (Warren et al. 1967).

#### *Araneusgrossus* (C. L. Koch, 1844)

**Distribution.** Europe to Central Asia (World Spider Catalog 2023).

**Recorded interactions with *H.cunea*.** South of the European part of former USSR (Sharov et al. 1984).

**Prey stage.** Larva (Sharov et al. 1984).

#### *Araneus* sp.

**Recorded interactions with *H.cunea*.** Japan [Fuchu, Akikawa (Kunimi 1983)].

**Prey stage.** Larva (Kunimi 1983).

**Notes.** Collected from fall webworm nest (Kunimi 1983).

#### *Araneusmacacus* Uyemura, 1961

**Distribution.** Russia (Far East), Japan (World Spider Catalog 2023).

**Recorded interactions with *H.cunea*.** Japan (Kayashima 1967 as *A.ventricosusmacacus*).

**Prey stage.** Pupa or adult (Kayashima 1967)

#### *Acacesiahamata* (Hentz, 1847)

**Distribution.** USA to Argentina (World Spider Catalog 2023).

**Recorded interactions with *H.cunea*.** USA [Arkansas (Warren et al. 1967)].

**Prey stage.** Larva (Warren et al. 1967).

**Notes.** Collected from fall webworm nest (Warren et al. 1967).

#### *Aoaraneuspentagrammicus* (Karsch, 1879)

**Distribution.** Korea, Japan, China (World Spider Catalog 2023).

**Recorded interactions with *H.cunea*.** Japan (Kayashima 1967 as *Araneuspentagrammicus*).

**Prey stage.** Pupa or adult (Kayashima 1967).

#### *Argiopeamoena* L. Koch, 1878

**Distribution.** China, Korea, Japan (World Spider Catalog 2023).

**Recorded interactions with *H.cunea*.** Japan (Kayashima 1967).

**Prey stage.** Pupa or adult (Kayashima 1967).

#### *Argiopebruennichii* (Scopoli, 1772)

**Distribution.** Europe, Turkey, Israel, Russia (Europe to Far East), Caucasus, Iran, Central Asia to China, Korea, Japan (World Spider Catalog 2023).

**Recorded interactions with *H.cunea*.** Japan (Kayashima 1967).

**Prey stage.** Pupa or adult (Kayashima 1967).

#### *Argiope* sp.

**Recorded interactions with *H.cunea*.** China [Liaocheng, Shandong Province (Yue et al. 2016)].

**Prey stage.** Larva (Yue et al. 2016).

#### *Bijoaraneusmitificus* (Simon, 1886)

**Distribution.** Pakistan, India, Bangladesh, China, Thailand, Cambodia, Singapore, Philippines, New Guinea (World Spider Catalog 2023).

**Recorded interactions with *H.cunea*.** Japan [Akikawa (Kunimi 1983 as *Araneusmitificus*)].

**Prey stage.** Larva (Kunimi 1983).

**Notes.** Collected from fall webworm nest (Kunimi 1983).

#### *Cyclosaatrata* Bösenberg & Strand, 1906

**Distribution.** China, Korea, Japan, Russia (World Spider Catalog 2023).

**Recorded interactions with *H.cunea*.** Japan (Kayashima 1967).

**Prey stage.** Pupa or adult (Kayashima 1967).

#### *Cyclosa* sp.

**Recorded interactions with *H.cunea*.** China [Liaocheng, Shandong Province (Yue et al. 2016)].

**Prey stage.** Larva (Yue et al. 2016).

#### *Epeira* sp.

**Recorded interactions with *H.cunea*.** USA [Arkansas (Warren et al. 1967)].

**Prey stage.** Larva (Warren et al. 1967).

**Notes.** Collected from fall webworm nest (Warren et al. 1967).

#### *Eustalaanastera* (Walckenaer, 1841)

**Distribution.** North and Central America (World Spider Catalog 2023).

**Recorded interactions with *H.cunea*.** USA [Arkansas (Warren et al. 1967)].

**Prey stage.** Larva (Warren et al. 1967).

**Notes.** Collected from fall webworm nest (Warren et al. 1967).

#### *Eustala* sp.

**Recorded interactions with *H.cunea*.** USA [Arkansas (Warren et al. 1967)].

**Prey stage.** Larva (Warren et al. 1967).

**Notes.** Collected from fall webworm nest (Warren et al. 1967).

#### *Gibbaraneabituberculata* (Walckenaer, 1802)

**Distribution.** North Africa, Europe, Turkey, Israel, Russia (Europe to Far East), Caucasus, Iran, Central Asia to China, Japan, India (World Spider Catalog 2023).

**Recorded interactions with *H.cunea*.** Italy [Italy [Po Valley (Groppali et al. 1993 as *Araneusbituberculatus*), Pavia (Camerini and Groppali 1999)].

**Prey stage.** Larva (Groppali et al. 1993; Camerini and Groppali 1999).

#### *Larinioidescornutus* (Clerck, 1757)

**Distribution.** North America, Europe, Turkey, Israel, Caucasus, Russia (Europe to Far East), Iran, China, Korea, Japan (World Spider Catalog 2023).

**Recorded interactions with *H.cunea*.** South of the European part of former USSR (Sharov et al. 1984 as *Nucteneacornuta*), Italy [Po Valley (Groppali et al. 1993)].

**Prey stage.** Larva (Sharov et al. 1984; Groppali et al. 1993).

#### *Larinioidespatagiatus* (Clerck, 1757)

**Distribution.** North America, Europe, Turkey, Caucasus, Russia (Europe to Far East), Central Asia, China, Mongolia, Japan (World Spider Catalog 2023).

**Recorded interactions with *H.cunea*.** South of the European part of former USSR (Sharov et al. 1984 as *Nucteneapatagiata*).

**Prey stage.** Larva (Sharov et al. 1984).

#### *Micrathenagracilis* (Walckenaer, 1805)

**Distribution.** North and Central America (World Spider Catalog 2023).

**Recorded interactions with *H.cunea*.** USA [Arkansas (Warren et al. 1967)].

**Prey stage.** Larva (Warren et al. 1967).

**Notes.** Collected from fall webworm nest (Warren et al. 1967).

#### *Neosconaarabesca* (Walckenaer, 1841)

**Distribution.** North, Central America, Caribbean. Introduced to Nepal, China (World Spider Catalog 2023).

**Recorded interactions with *H.cunea*.** USA [Arkansas (Warren et al. 1967)].

**Prey stage.** Larva (Warren et al. 1967).

**Notes.** Collected from fall webworm nest (Warren et al. 1967).

#### *Neosconacruciferabenjamina* (Lucas, 1838)

**Distribution.** North America. Introduced to Hawaii, Canary Is., Madeira (World Spider Catalog 2023).

**Recorded interactions with *H.cunea*.** USA [Arkansas (Warren et al. 1967 as *N.sacra*)].

**Prey stage.** Larva (Warren et al. 1967).

**Notes.** Collected from fall webworm nest (Warren et al. 1967).

#### *Neosconapratensis* (Hentz, 1847)

**Distribution.** Canada, USA (World Spider Catalog 2023).

**Recorded interactions with *H.cunea*.** USA [Arkansas (Warren et al. 1967)].

**Prey stage.** Larva (Warren et al. 1967).

**Notes.** Collected from fall webworm nest (Warren et al. 1967).

#### *Neosconascylloides* (Bösenberg & Strand, 1906)

**Distribution.** Russia (Far East), Korea, Japan, China (World Spider Catalog 2023).

**Recorded interactions with *H.cunea*.** Japan (Kayashima 1967).

**Prey stage.** Pupa or adult (Kayashima 1967).

#### *Ocrepeiraectypa* (Walckenaer, 1841)

**Distribution.** USA (World Spider Catalog 2023).

**Recorded interactions with *H.cunea*.** USA [Arkansas (Warren et al. 1967 as *Wixiaectypa*)].

**Prey stage.** Larva (Warren et al. 1967).

**Notes.** Collected from fall webworm nest (Warren et al. 1967).

#### *Singahamata* (Clerck, 1757)

**Distribution.** Europe, Turkey, Russia (Europe to Far East), Caucasus to Central Asia, China, Korea, Japan (World Spider Catalog 2023).

**Recorded interactions with *H.cunea*.** Italy [Central Padana Plain (Groppali et al. 1994)].

**Prey stage.** Larva (Groppali et al. 1994).

#### *Singa* sp.

**Recorded interactions with *H.cunea*.** USA [Arkansas (Warren et al. 1967)].

**Prey stage.** Larva (Warren et al. 1967).

**Notes.** Collected from fall webworm nest (Warren et al. 1967).

#### *Yaginumiasia* (Strand, 1906)

**Distribution.** China, Korea, Japan (World Spider Catalog 2023).

**Recorded interactions with *H.cunea*.** Japan [Akikawa (Kunimi 1983 as *Y.zea*)].

**Prey stage.** Larva (Kunimi 1983).

**Notes.** Collected from fall webworm nest (Kunimi 1983).

##### ﻿﻿Cheiracanthiidae 红螯蛛科

#### *Cheiracanthiumerraticum* (Walckenaer, 1802)

**Distribution.** Azores, Europe, Turkey, Caucasus, Russia (Europe to Far East), Iran, Central Asia, China, Korea, Japan (World Spider Catalog 2023).

**Recorded interactions with *H.cunea*.** South of the European part of former USSR (Sharov et al. 1984).

**Prey stage.** Larva (Sharov et al. 1984).

#### *Cheiracanthiumeutittha* Bösenberg & Strand, 1906

**Distribution.** China (Taiwan), Japan (World Spider Catalog 2023).

**Recorded interactions with *H.cunea*.** Japan [Fuchu, Akikawa (Kunimi 1983 as *Chiracanthiumeutittha*)].

**Prey stage.** Larva (Kunimi 1983).

**Notes.** Collected from fall webworm nest (Kunimi 1983).

#### *Cheiracanthiuminclusum* (Hentz, 1847)

**Distribution.** North America, Central America, Caribbean, South America. Introduced to Réunion (World Spider Catalog 2023).

**Recorded interactions with *H.cunea*.** USA [Arkansas (Warren et al. 1967 as *Chiracanthiuminclusum*)].

**Prey stage.** Larva (Warren et al. 1967).

**Notes.** Collected from fall webworm nest (Warren et al. 1967).

#### *Cheiracanthiumlascivum* Karsch, 1879

**Distribution.** Russia (Sakhalin), China, Korea, Japan (World Spider Catalog 2023).

**Recorded interactions with *H.cunea*.** Japan [Akikawa (Kunimi 1983 as *Chiracanthiumlascivum*)].

**Prey stage.** Larva (Kunimi 1983).

**Notes.** Collected from fall webworm nest (Kunimi 1983).

#### *Cheiracanthiummildei* L. Koch, 1864

**Distribution.** Azores, Europe, North Africa, Turkey, Middle East, Caucasus, Russia (Europe) to Central Asia. Introduced to North America, Argentina (World Spider Catalog 2023).

**Recorded interactions with *H.cunea*.** Italy [Po Valley (Groppali et al. 1993)].

**Prey stage.** Larva (Groppali et al. 1993).

#### *Cheiracanthiumjaponicum* Bösenberg & Strand, 1906

**Distribution.** Russia (Far East), Mongolia, China, Korea, Japan (World Spider Catalog 2023).

**Recorded interactions with *H.cunea*.** Japan [Fuchu (Kunimi 1983 as *Chiracanthiumjaponicum*)].

**Prey stage.** Larva (Kunimi 1983).

**Notes.** Collected from fall webworm nest (Kunimi 1983).

#### *Chieracanthium* sp.

**Recorded interactions with *H.cunea*.** Italy [Po Valley (Groppali et al. 1993)].

**Prey stage.** Larva (Groppali et al. 1993).

#### *Cheiracanthiumunicum* Bösenberg & Strand, 1906

**Distribution.** Korea, Japan, China, Laos (World Spider Catalog 2023).

**Recorded interactions with *H.cunea*.** Japan [Akikawa (Kunimi 1983 as *Chiracanthiumunicum*)].

**Prey stage.** Larva (Kunimi 1983).

**Notes.** Collected from fall webworm nest (Kunimi 1983).

##### ﻿﻿Clubionidae 管巢蛛科

#### *Buclionajucunda* (Karsch, 1879)

**Distribution.** Russia (Far East), Korea, Japan, China (World Spider Catalog 2023).

**Recorded interactions with *H.cunea*.** Japan [Fuchu (Kunimi 1983 as *Clubionajucunda*)].

**Prey stage.** Larva (Kunimi 1983).

**Notes.** Collected from fall webworm nest (Kunimi 1983).

#### *Clubionaabboti* L. Koch, 1866

**Distribution.** Canada, USA (World Spider Catalog 2023).

**Recorded interactions with *H.cunea*.** USA [Arkansas (Warren et al. 1967)].

**Prey stage.** Larva (Warren et al. 1967).

**Notes.** Collected from fall webworm nest (Warren et al. 1967).

#### *Clubionacorrugata* Bösenberg & Strand, 1906

**Distribution.** Russia (Far East), China, Korea, Japan, Thailand (World Spider Catalog 2023).

**Recorded interactions with *H.cunea*.** Japan [Fuchu (Kunimi 1983)].

**Prey stage.** Larva (Kunimi 1983).

**Notes.** Collected from fall webworm nest (Kunimi 1983).

#### *Clubionadiversa* O. Pickard-Cambridge, 1862

**Distribution.** Europe, Caucasus, Russia (Europe to Far East), Kazakhstan, Pakistan, Korea, Japan (World Spider Catalog 2023).

**Recorded interactions with *H.cunea*.** USA [Arkansas (Warren et al. 1967 as *Clubionapallens*)].

**Prey stage.** Larva (Warren et al. 1967).

**Notes.** Collected from fall webworm nest (Warren et al. 1967).

#### *Clubionalutescens* Westring, 1851

**Distribution.** Europe, Turkey, Caucasus, Russia (Europe to Far East), Iran, Kazakhstan, Korea, Japan. Introduced to North America (World Spider Catalog 2023).

**Recorded interactions with *H.cunea*.** Japan [Akikawa (Kunimi 1983)].

**Prey stage.** Larva (Kunimi 1983).

**Notes.** Collected from fall webworm nest (Kunimi 1983).

#### *Clubionamarmorata* L. Koch, 1866

**Distribution.** France to Ukraine and Turkey (World Spider Catalog 2023).

**Recorded interactions with *H.cunea*.** South of the European part of former USSR (Sharov et al. 1984).

**Prey stage.** Larva (Sharov et al. 1984).

#### *Clubionamoesta* Banks, 1896

**Distribution.** USA, Canada, China (World Spider Catalog 2023).

**Recorded interactions with *H.cunea*.** USA [Arkansas (Warren et al. 1967)].

**Prey stage.** Larva (Warren et al. 1967).

**Notes.** Collected from fall webworm nest (Warren et al. 1967).

#### *Clubionaobesa* Hentz, 1847

**Distribution.** USA, Canada (World Spider Catalog 2023).

**Recorded interactions with *H.cunea*.** USA [Arkansas (Warren et al. 1967)].

**Prey stage.** Larva (Warren et al. 1967).

**Notes.** Collected from fall webworm nest (Warren et al. 1967).

#### *Clubionapallidula* (Clerck, 1757)

**Distribution.** Europe, Caucasus, Russia (Europe to Far East), Central Asia. Introduced to North America (World Spider Catalog 2023).

**Recorded interactions with *H.cunea*.** South of the European part of former USSR (Sharov et al. 1984).

**Prey stage.** Larva (Sharov et al. 1984).

#### *Clubiona* sp.

**Recorded interactions with *H.cunea*.** USA [Arkansas (Warren et al. 1967)].

**Prey stage.** Larva (Warren et al. 1967).

**Notes.** Collected from fall webworm nest (Warren et al. 1967).

#### *Clubionavigil* Karsch, 1879

**Distribution.** Russia (Kurile Is.), Korea, Japan, China (World Spider Catalog 2023).

**Recorded interactions with *H.cunea*.** Japan [Fuchu, Akikawa (Kunimi 1983)].

**Prey stage.** Larva (Kunimi 1983).

**Notes.** Collected from fall webworm nest (Kunimi 1983).

#### *Elaverexcepta* (L. Koch, 1866)

**Distribution.** Canada, USA, Caribbean (World Spider Catalog 2023).

**Recorded interactions with *H.cunea*.** USA [Arkansas (Warren et al. 1967 as *Clubionidesexcepta*)].

**Prey stage.** Larva (Warren et al. 1967).

**Notes.** Collected from fall webworm nest (Warren et al. 1967).

##### ﻿﻿Dictynidae 叶蛛科

#### *Dictynaarundinacea* (Linnaeus, 1758)

**Distribution.** North America, Europe, Turkey, Caucasus, Russia (Europe to Far East), Iran, Central Asia, China, Korea, Japan (World Spider Catalog 2023).

**Recorded interactions with *H.cunea*.** Moldova (Sharov et al. 1984).

**Prey stage.** Larva (Sharov et al. 1984).

#### *Dictynafoliicola* Bösenberg & Strand, 1906

**Distribution.** Russia (Far East), China, Korea, Japan (World Spider Catalog 2023).

**Recorded interactions with *H.cunea*.** Japan (Kayashima 1967).

**Prey stage.** Larva (Kayashima 1967).

#### *Dictynapusilla* Thorell, 1856

**Distribution.** Europe, Turkey, Caucasus, Russia (Europe to Far East), Central Asia (World Spider Catalog 2023).

**Recorded interactions with *H.cunea*.** Italy [Po Valley (Groppali et al. 1993), Central Padana Plain (Groppali et al. 1994)].

**Prey stage.** Larva (Groppali et al. 1994).

#### *Dictyna* sp.

**Recorded interactions with *H.cunea*.** USA [Arkansas (Warren et al. 1967)].

**Prey stage.** Larva (Warren et al. 1967).

**Notes.** Collected from fall webworm nest (Warren et al. 1967).

#### *Phantynasegregata* (Gertsch & Mulaik, 1936)

**Distribution.** USA, Mexico (World Spider Catalog 2023).

**Recorded interactions with *H.cunea*.** USA [Arkansas (Warren et al. 1967 as Dictynaaffsegregata)].

**Prey stage.** Larva (Warren et al. 1967).

**Notes.** Collected from fall webworm nest (Warren et al. 1967).

##### ﻿﻿Gnaphosidae 平腹蛛科

#### *Cesoniabilineata* (Hentz, 1847)

**Distribution.** Canada, USA, Mexico (World Spider Catalog 2023).

**Recorded interactions with *H.cunea*.** USA [Arkansas (Warren et al. 1967)].

**Prey stage.** Larva (Warren et al. 1967).

**Notes.** Collected from fall webworm nest (Warren et al. 1967).

#### *Drassyllus* sp.

**Recorded interactions with *H.cunea*.** USA [Arkansas (Warren et al. 1967)].

**Prey stage.** Larva (Warren et al. 1967).

**Notes.** Collected from fall webworm nest (Warren et al. 1967).

#### *Zelotes* sp.

**Recorded interactions with *H.cunea*.** USA [Arkansas (Warren et al. 1967)].

**Prey stage.** Larva (Warren et al. 1967).

**Notes.** Collected from fall webworm nest (Warren et al. 1967).

##### ﻿﻿Linyphiidae 皿蛛科

#### *Frontinellinafrutetorum* (C. L. Koch, 1835)

**Distribution.** Europe, North Africa, Turkey, Caucasus, Russia (Europe to south Siberia), Iran, Kazakhstan, Central Asia (World Spider Catalog 2023).

**Recorded interactions with *H.cunea*.** Italy [Po Valley (Groppali et al. 1993)].

**Prey stage.** Larva (Groppali et al. 1993).

#### *Grammonotamaculata* Banks, 1896

**Distribution.** USA, Costa Rica (World Spider Catalog 2023).

**Recorded interactions with *H.cunea*.** USA [Arkansas (Warren et al. 1967)].

**Prey stage.** Larva (Warren et al. 1967).

**Notes.** Collected from fall webworm nest (Warren et al. 1967).

#### *Linyphia* sp.

**Recorded interactions with *H.cunea*.** South of the European part of former USSR (Sharov et al. 1984).

**Prey stage.** Larva (Sharov et al. 1984).

#### *Oedothoraxretusus* (Westring, 1851)

**Distribution.** Europe, Turkey, Caucasus, Russia (Europe to north-eastern Siberia), Kazakhstan, China (World Spider Catalog 2023).

**Recorded interactions with *H.cunea*.** China [Dandong City, Liaoning Province (Shu and Yu 1985)].

**Prey stage.** Larva (Shu and Yu 1985).

#### *Strandellaquadrimaculata* (Uyemura, 1937)

**Distribution.** Japan (World Spider Catalog 2023).

**Recorded interactions with *H.cunea*.** Japan [Fuchu (Kunimi 1983].

**Prey stage.** Larva (Kunimi 1983).

**Notes.** Collected from fall webworm nest (Kunimi 1983).

##### ﻿﻿Lycosidae 狼蛛科

#### *Arctosacinerea* (Fabricius, 1777)

**Distribution.** Europe, North Africa, Congo, Caucasus, Russia (Europe to Far East), Middle East, Kazakhstan, China, Korea, Japan (World Spider Catalog 2023).

**Recorded interactions with *H.cunea*.** South of the European part of former USSR (Sharov et al. 1984).

**Prey stage.** Larva (Sharov et al. 1984).

#### *Pardosaastrigera* L. Koch, 1878

**Distribution.** Russia (Far East), Korea, Japan, China (World Spider Catalog 2023).

**Recorded interactions with *H.cunea*.** Japan (Kayashima 1967 as *Lycosat-insignita*).

**Prey stage.** Larva and adult (Kayashima 1967).

##### ﻿﻿Mimetidae 拟态蛛科

#### *Mimetuspuritanus* Chamberlin, 1923

**Distribution.** USA, Jamaica (World Spider Catalog 2023).

**Recorded interactions with *H.cunea*.** USA [Arkansas (Warren et al. 1967)].

**Prey stage.** Larva (Warren et al. 1967).

**Notes.** Collected from fall webworm nest (Warren et al. 1967).

#### *Mimetussyllepsicus* Hentz, 1832

**Distribution.** USA, Mexico (World Spider Catalog 2023).

**Recorded interactions with *H.cunea*.** USA [Arkansas (Warren et al. 1967 as *M.interfector*)].

**Prey stage.** Larva (Warren et al. 1967).

**Notes.** Collected from fall webworm nest (Warren et al. 1967).

##### ﻿﻿Nephilidae 芥蛛科

#### *Trichonephilaclavata* (L. Koch, 1878)

**Distribution.** China, Korea, Japan (World Spider Catalog 2023).

**Recorded interactions with *H.cunea*.** Japan (Kayashima 1967 as *Nephilaclavata*), Japan [Akikawa (Kunimi 1983 as *Nephilaclavata*)].

**Prey stage.** Larva (Kunimi 1983) pupa or adult (Kayashima 1967).

**Notes.** Collected from fall webworm nest (Kunimi 1983).

##### ﻿﻿Oecobiidae 壁钱科

#### *Oecobiuscellariorum* (Dugès, 1836)

**Distribution.** Mediterranean, Russia (Europe), Azerbaijan, Jordan, Iran; introduced to USA, China, Japan (World Spider Catalog 2023).

**Recorded interactions with *H.cunea*.** USA [Arkansas (Warren et al. 1967 as *Oecobiustexanus*)].

**Prey stage.** Larva (Warren et al. 1967).

**Notes.** Collected from fall webworm nest (Warren et al. 1967).

#### *Uroctealesserti* Schenkel, 1936

**Distribution.** China, Korea (World Spider Catalog 2023).

**Recorded interactions with *H.cunea*.** China [Dandong City, Liaoning Province (Shu and Yu 1985)].

**Prey stage.** Larva (Shu and Yu 1985).

##### ﻿﻿Philodromidae 逍遥蛛科

#### *Philodromusabbotii* Walckenaer, 1837, nomen dubium

**Distribution.** USA (World Spider Catalog 2023).

**Recorded interactions with *H.cunea*.** USA [Arkansas (Warren et al. 1967)].

**Prey stage.** Larva (Warren et al. 1967).

**Notes.** Collected from fall webworm nest (Warren et al. 1967).

#### *Philodromusaureolus* (Clerck, 1757)

**Distribution.** Europe, Turkey, Caucasus, Russia (Europe to Central Asia and Middle Siberia), Kazakhstan, Iran, Central Asia, Mongolia, China, Korea, Japan (World Spider Catalog 2023).

**Recorded interactions with *H.cunea*.** South of the European part of former USSR (Sharov et al. 1984), Italy [Po Valley (Groppali et al. 1993), Central Padana Plain (Groppali et al. 1994)].

**Prey stage.** Larva (Sharov et al. 1984; Groppali et al. 1994).

#### *Philodromuscespitum* (Walckenaer, 1802)

**Distribution.** North America, Europe, North Africa, Turkey, Caucasus, Russia (Europe to Far East), Kazakhstan, Iran, Mongolia, China, Korea, Japan (World Spider Catalog 2023).

**Recorded interactions with *H.cunea*.** Italy [Po Valley (Groppali et al. 1993 as *P.caespitum*)].

**Prey stage.** Larva (Groppali et al. 1993).

#### *Philodromuskeyserlingi* Marx, 1890

**Distribution.** USA, Canada (World Spider Catalog 2023).

**Recorded interactions with *H.cunea*.** USA [Arkansas (Warren et al. 1967)].

**Prey stage.** Larva (Warren et al. 1967).

**Notes.** Collected from fall webworm nest (Warren et al. 1967).

#### *Philodromusmarxii* Keyserling, 1884

**Distribution.** USA (World Spider Catalog 2023).

**Recorded interactions with *H.cunea*.** USA [Arkansas (Warren et al. 1967)].

**Prey stage.** Larva (Warren et al. 1967).

**Notes.** Collected from fall webworm nest (Warren et al. 1967).

#### *Philodromuspernix* Blackwall, 1846

**Distribution.** USA, Canada (World Spider Catalog 2023).

**Recorded interactions with *H.cunea*.** USA [Arkansas (Warren et al. 1967)].

**Prey stage.** Larva (Warren et al. 1967).

**Notes.** Collected from fall webworm nest (Warren et al. 1967).

#### *Philodromusrufus* Walckenaer, 1826

**Distribution.** North America, Europe, Turkey, Caucasus, Russia (Europe to Far East), Kazakhstan, Iran, Central Asia, Mongolia, China, Korea, Japan (World Spider Catalog 2023).

**Recorded interactions with *H.cunea*.** USA [Arkansas (Warren et al. 1967)].

**Prey stage.** Larva (Warren et al. 1967).

**Notes.** Collected from fall webworm nest (Warren et al. 1967).

#### *Philodromus* sp.

**Recorded interactions with *H.cunea*.** USA [Arkansas (Warren et al. 1967)].

**Prey stage.** Larva (Warren et al. 1967).

**Notes.** Collected from fall webworm nest (Warren et al. 1967).

#### *Philodromus* sp.

**Recorded interactions with *H.cunea*.** Italy [Po Valley (Groppali et al. 1993)].

**Prey stage.** Larva (Groppali et al. 1993).

#### *Philodromusspinitarsis* Simon, 1895

**Distribution.** Russia (south Siberia, Far East), China, Korea, Japan (World Spider Catalog 2023).

**Recorded interactions with *H.cunea*.** China [Dandong City, Liaoning Province (Shu and Yu 1985)].

**Prey stage.** Larva (Shu and Yu 1985).

#### *Philodromusvulgaris* (Hentz, 1847)

**Distribution.** USA, Canada (World Spider Catalog 2023).

**Recorded interactions with *H.cunea*.** USA [Arkansas (Warren et al. 1967)].

**Prey stage.** Larva (Warren et al. 1967).

**Notes.** Collected from fall webworm nest (Warren et al. 1967).

#### *Thanatusformicinus* (Clerck, 1757)

**Distribution.** North America, Europe, North Africa, Turkey, Caucasus, Russia (Europe to Far East), Iraq, Iran, Kazakhstan, Central Asia, China, Japan (World Spider Catalog 2023).

**Recorded interactions with *H.cunea*.** USA [Arkansas (Tadić 1963 as *Philodromusformicinus*)].

**Prey stage.** Larva (Tadić 1963).

##### ﻿﻿Pisauridae 盗蛛科

#### *Pisauramirabilis* (Clerck, 1757)

**Distribution.** Europe, Turkey, Middle East, Caucasus, Russia (Europe to Middle Siberia), Central Asia, China (World Spider Catalog 2023).

**Recorded interactions with *H.cunea*.** South of the European part of former USSR (Sharov et al. 1984).

**Prey stage.** Larva (Sharov et al. 1984).

#### *Pisauralama* Bösenberg & Strand, 1906

**Distribution.** Russia (Far East), China, Korea, Japan (World Spider Catalog 2023).

**Recorded interactions with *H.cunea*.** Japan (Kayashima 1967).

**Prey stage.** Pupa or adult (Kayashima 1967).

#### *Pisaurinamira* (Walckenaer, 1837)

**Distribution.** Canada, USA (World Spider Catalog 2023).

**Recorded interactions with *H.cunea*.** USA [Arkansas (Warren et al. 1967 as *Dapanusmirus*)].

**Prey stage.** Larva (Warren et al. 1967).

**Notes.** Collected from fall webworm nest (Warren et al. 1967).

##### ﻿﻿Salticidae 跳蛛科

#### *Aelurillusv-insignitus* (Clerck, 1757)

**Distribution.** Europe, Turkey, Caucasus, Russia (Europe to Far East), Kazakhstan, Central Asia, China (World Spider Catalog 2023).

**Recorded interactions with *H.cunea*.** Italy [Central Padana Plain (Groppali et al. 1994)].

**Prey stage.** Larva (Groppali et al. 1994).

#### *Carrhotusxanthogramma* (Latreille, 1819)

**Distribution.** Europe, Turkey, Caucasus, Russia (Europe to Far East), China, Korea, Japan (World Spider Catalog 2023).

**Recorded interactions with *H.cunea*.** Japan [Akikawa (Kunimi 1983)].

**Prey stage.** Larva (Kunimi 1983).

**Notes.** Collected from fall webworm nest (Kunimi 1983).

#### *Colonussylvanus* (Hentz, 1846)

**Distribution.** USA to Panama (World Spider Catalog 2023).

**Recorded interactions with *H.cunea*.** USA [Arkansas (Warren et al. 1967 as *Thiodinasylvana*)].

**Prey stage.** Larva (Warren et al. 1967).

**Notes.** Collected from fall webworm nest (Warren et al. 1967).

#### *Erismilitaris* (Hentz, 1845)

**Distribution.** USA, Canada (World Spider Catalog 2023).

**Recorded interactions with *H.cunea*.** USA [Arkansas (Warren et al. 1967 as *E.marginatus*)].

**Prey stage.** Larva (Warren et al. 1967).

**Notes.** Collected from fall webworm nest (Warren et al. 1967).

#### *Erisrufa* (C. L. Koch, 1846)

**Distribution.** USA (World Spider Catalog 2023).

**Recorded interactions with *H.cunea*.** USA [Arkansas (Warren et al. 1967 as *E.pineus*)].

**Prey stage.** Larva (Warren et al. 1967).

**Notes.** Collected from fall webworm nest (Warren et al. 1967).

#### *Eris* sp.

**Recorded interactions with *H.cunea*.** USA [Arkansas (Warren et al. 1967)].

**Prey stage.** Larva (Warren et al. 1967).

**Notes.** Collected from fall webworm nest (Warren et al. 1967).

#### *Evarchaarcuata* (Clerck, 1757)

**Distribution.** Europe, Turkey, Caucasus, Russia (Europe to Far East), Kazakhstan, Iran, Central Asia, China, Japan (World Spider Catalog 2023).

**Recorded interactions with *H.cunea*.** South of the European part of former USSR (Sharov et al. 1984).

**Prey stage.** Larva (Sharov et al. 1984).

#### *Evarchafalcata* (Clerck, 1757)

**Distribution.** Europe, Turkey, Caucasus, Russia (Europe to south Siberia), Kazakhstan, Afghanistan, China (World Spider Catalog 2023).

**Recorded interactions with *H.cunea*.** South of the European part of former USSR (Sharov et al. 1984).

**Prey stage.** Larva (Sharov et al. 1984).

#### *Heliophanusauratus* C. L. Koch, 1835

**Distribution.** Europe, Turkey, Caucasus, Russia (Europe to south Siberia), Kazakhstan, Central Asia, China (World Spider Catalog 2023).

**Recorded interactions with *H.cunea*.** South of the European part of former USSR (Sharov et al. 1984).

**Prey stage.** Larva (Sharov et al. 1984).

#### *Hentziamitrata* (Hentz, 1846)

**Distribution.** USA, Canada, Bahama Is. (World Spider Catalog 2023).

**Recorded interactions with *H.cunea*.** USA [Arkansas (Warren et al. 1967)].

**Prey stage.** Larva (Warren et al. 1967).

**Notes.** Collected from fall webworm nest (Warren et al. 1967).

#### *Hentziapalmarum* (Hentz, 1832)

**Distribution.** North America, Bermuda, Bahama Is., Cuba (World Spider Catalog 2023).

**Recorded interactions with *H.cunea*.** USA [Arkansas (Warren et al. 1967)].

**Prey stage.** Larva (Warren et al. 1967).

**Notes.** Collected from fall webworm nest (Warren et al. 1967).

#### *Hentzia* sp.

**Recorded interactions with *H.cunea*.** USA [Arkansas (Warren et al. 1967)].

**Prey stage.** Larva (Warren et al. 1967).

**Notes.** Collected from fall webworm nest (Warren et al. 1967).

#### *Icius* sp.

**Recorded interactions with *H.cunea*.** USA [Arkansas (Warren et al. 1967)].

**Prey stage.** Larva (Warren et al. 1967).

**Notes.** Collected from fall webworm nest (Warren et al. 1967).

#### *Jotus* sp.

**Recorded interactions with *H.cunea*.** Japan [Fuchu, Akikawa (Kunimi 1983)].

**Prey stage.** Larva (Kunimi 1983).

**Notes.** Collected from fall webworm nest (Kunimi 1983).

#### *Macaroerisnidicolens* (Walckenaer, 1802)

**Distribution.** Macaronesia, Europe, North Africa to Turkey, Caucasus, Turkmenistan, Iran; introduced to Sri Lanka (World Spider Catalog 2023).

**Recorded interactions with *H.cunea*.** Italy [Po Valley (Groppali et al. 1993 as *Erisnidicolens*), Central Padana Plain (Groppali et al. 1994 as *Erisnidicolens*)].

**Prey stage.** Larva (Groppali et al. 1994).

#### *Maeviainclemens* (Walckenaer, 1837)

**Distribution.** USA, Canada (World Spider Catalog 2023).

**Recorded interactions with *H.cunea*.** USA [Arkansas (Warren et al. 1967 as *M.vittata*)].

**Prey stage.** Larva (Warren et al. 1967).

**Notes.** Collected from fall webworm nest (Warren et al. 1967).

#### *Metacyrbataeniola* (Hentz, 1846)

**Distribution.** USA, Mexico (World Spider Catalog 2023).

**Recorded interactions with *H.cunea*.** USA [Arkansas (Warren et al. 1967)].

**Prey stage.** Larva (Warren et al. 1967).

**Notes.** Collected from fall webworm nest (Warren et al. 1967).

#### *Metaphidippus* sp.

**Recorded interactions with *H.cunea*.** USA [Arkansas (Warren et al. 1967)].

**Prey stage.** Larva (Warren et al. 1967).

**Notes.** Collected from fall webworm nest (Warren et al. 1967).

#### *Myrmarachnejaponica* (Karsch, 1879)

**Distribution.** Russia (Far East), China, Korea, Japan (World Spider Catalog 2023).

**Recorded interactions with *H.cunea*.** Japan [Akikawa (Kunimi 1983)].

**Prey stage.** Larva (Kunimi 1983).

**Notes.** Collected from fall webworm nest (Kunimi 1983).

#### *Orienticiusvulpes* (Grube, 1861)

**Distribution.** Russia (south Siberia to Far East), China, Korea, Japan (World Spider Catalog 2023).

**Recorded interactions with *H.cunea*.** Japan [Akikawa (Kunimi 1983 as *Dendryphantesatratus*)].

**Prey stage.** Larva (Kunimi 1983).

**Notes.** Collected from fall webworm nest (Kunimi 1983).

#### *Paraphidippusaurantius* (Lucas, 1833)

**Distribution.** USA to Panama, Greater Antilles (World Spider Catalog 2023).

**Recorded interactions with *H.cunea*.** USA [Arkansas (Warren et al. 1967 as *Erisaurantia*)].

**Prey stage.** Larva (Warren et al. 1967).

**Notes.** Collected from fall webworm nest (Warren et al. 1967).

#### *Peckhamiapicata* (Hentz, 1846)

**Distribution.** North America (World Spider Catalog 2023).

**Recorded interactions with *H.cunea*.** USA [Arkansas (Warren et al. 1967)].

**Prey stage.** Larva (Warren et al. 1967).

**Notes.** Collected from fall webworm nest (Warren et al. 1967).

#### *Pelegrinagalathea* (Walckenaer, 1837)

**Distribution.** Canada to Costa Rica, Bermuda (World Spider Catalog 2023).

**Recorded interactions with *H.cunea*.** USA [Arkansas (Warren et al. 1967 as *Metaphidippusgalathea*)].

**Prey stage.** Larva (Warren et al. 1967).

**Notes.** Collected from fall webworm nest (Warren et al. 1967).

#### *Pelegrinainsignis* (Banks, 1892)

**Distribution.** USA, Canada (World Spider Catalog 2023).

**Recorded interactions with *H.cunea*.** USA [Arkansas (Warren et al. 1967 as *Metaphidippusinsignis*)].

**Prey stage.** Larva (Warren et al. 1967).

**Notes.** Collected from fall webworm nest (Warren et al. 1967).

#### *Pelegrinaproterva* (Walckenaer, 1837)

**Distribution.** USA, Canada (World Spider Catalog 2023).

**Recorded interactions with *H.cunea*.** USA [Arkansas (Warren et al. 1967 as *Metaphidippusprotervus*)].

**Prey stage.** Larva (Warren et al. 1967).

**Notes.** Collected from fall webworm nest (Warren et al. 1967).

#### *Phintellacastriesiana* (Grube, 1861)

**Distribution.** Canary Is., Southern Europe, North Africa, Middle East, Turkey, Caucasus, Iran, Russia (Far East), Korea, Japan (World Spider Catalog 2023).

**Recorded interactions with *H.cunea*.** South of the European part of former USSR (Sharov et al. 1984 as *Iciuscastriesianus*).

**Prey stage.** Larva (Sharov et al. 1984).

#### *Platycryptusundatus* (De Geer, 1778)

**Distribution.** North America (World Spider Catalog 2023).

**Recorded interactions with *H.cunea*.** USA [Arkansas (Warren et al. 1967 as *Metacyrbaundata*)].

**Prey stage.** Larva (Warren et al. 1967).

**Notes.** Collected from fall webworm nest (Warren et al. 1967).

#### *Plexippoidesdoenitzi* (Karsch, 1879)

**Distribution.** China, Korea, Japan (World Spider Catalog 2023).

**Recorded interactions with *H.cunea*.** Japan (Kayashima 1967 as *Hasariusdoenitzi*).

**Prey stage.** Larva (Kayashima 1967).

#### *Plexippuspaykulli* (Audouin, 1826)

**Distribution.** Africa. Introduced to both Americas, Europe, Middle East, Nepal, southern Asia, Australia, Pacific Is. (World Spider Catalog 2023).

**Recorded interactions with *H.cunea*.** Japan (Kayashima 1967).

**Prey stage.** Larva (Kayashima 1967).

#### *Plexippus* sp.

**Recorded interactions with *H.cunea*.** USA [Arkansas (Warren et al. 1967)].

**Prey stage.** Larva (Warren et al. 1967).

**Notes.** Collected from fall webworm nest (Warren et al. 1967).

#### *Phidippusaudax* (Hentz, 1845)

**Distribution.** North America; introduced to Hawaii, Azores, India (World Spider Catalog 2023).

**Recorded interactions with *H.cunea*.** USA [Arkansas (Warren et al. 1967)].

**Prey stage.** Larva (Warren et al. 1967).

**Notes.** Collected from fall webworm nest (Warren et al. 1967).

#### *Phidippuscarolinensis* G. W. Peckham & E. G. Peckham, 1909

**Distribution.** USA, Mexico (World Spider Catalog 2023).

**Recorded interactions with *H.cunea*.** USA [Arkansas (Warren et al. 1967)].

**Prey stage.** Larva (Warren et al. 1967).

**Notes.** Collected from fall webworm nest (Warren et al. 1967).

#### *Phidippusclarus* Keyserling, 1885

**Distribution.** North America (World Spider Catalog 2023).

**Recorded interactions with *H.cunea*.** USA [Arkansas (Warren et al. 1967)].

**Prey stage.** Larva (Warren et al. 1967).

**Notes.** Collected from fall webworm nest (Warren et al. 1967).

#### *Phidippusinsignarius* C. L. Koch, 1846

**Distribution.** USA (World Spider Catalog 2023).

**Recorded interactions with *H.cunea*.** USA [Arkansas (Warren et al. 1967)].

**Prey stage.** Larva (Warren et al. 1967).

**Notes.** Collected from fall webworm nest (Warren et al. 1967).

#### *Phidippusmystaceus* (Hentz, 1846)

**Distribution.** USA (World Spider Catalog 2023).

**Recorded interactions with *H.cunea*.** USA [Arkansas (Warren et al. 1967 as *P.incertus*)].

**Prey stage.** Larva (Warren et al. 1967).

**Notes.** Collected from fall webworm nest (Warren et al. 1967).

#### *Phidippusprinceps* (G. W. Peckham & E. G. Peckham, 1883)

**Distribution.** USA, Canada (World Spider Catalog 2023).

**Recorded interactions with *H.cunea*.** USA [Arkansas (Warren et al. 1967)].

**Prey stage.** Larva (Warren et al. 1967).

**Notes.** Collected from fall webworm nest (Warren et al. 1967).

#### *Phidippusputnami* (G. W. Peckham & E. G. Peckham, 1883)

**Distribution.** USA (World Spider Catalog 2023).

**Recorded interactions with *H.cunea*.** USA [Arkansas (Warren et al. 1967)].

**Prey stage.** Larva (Warren et al. 1967).

**Notes.** Collected from fall webworm nest (Warren et al. 1967).

#### *Phidippuswhitmani* G. W. Peckham & E. G. Peckham, 1909

**Distribution.** USA, Canada (World Spider Catalog 2023).

**Recorded interactions with *H.cunea*.** USA [Arkansas (Warren et al. 1967)].

**Prey stage.** Larva (Warren et al. 1967).

**Notes.** Collected from fall webworm nest (Warren et al. 1967).

#### *Rheneatrata* (Karsch, 1881)

**Distribution.** Russia (south Siberia, Far East), China, Korea, Japan (World Spider Catalog 2023).

**Recorded interactions with *H.cunea*.** Japan [Fuchu, Akikawa (Kunimi 1983 as *Dendryphantesatratus*)].

**Prey stage.** Larva (Kunimi 1983).

**Notes.** Collected from fall webworm nest (Kunimi 1983).

#### *Salticuszebraneus* (C. L. Koch, 1837)

**Distribution.** Europe, Turkey, Russia (Europe, Caucasus), Iran (World Spider Catalog 2023).

**Recorded interactions with *H.cunea*.** South of the European part of former USSR (Sharov et al. 1984), Italy [Central Padana Plain (Groppali et al. 1994)].

**Prey stage.** Larva (Sharov et al. 1984; Groppali et al. 1994).

#### *Sassacuscyaneus* (Hentz, 1846)

**Distribution.** USA (World Spider Catalog 2023).

**Recorded interactions with *H.cunea*.** USA [Arkansas (Warren et al. 1967 as *Agassacyanea*)].

**Prey stage.** Larva (Warren et al. 1967).

**Notes.** Collected from fall webworm nest (Warren et al. 1967).

#### *Sibianorpullus* (Bösenberg & Strand, 1906)

**Distribution.** Russia (Far East), Korea, Japan, China (World Spider Catalog 2023).

**Recorded interactions with *H.cunea*.** Japan [Akikawa (Kunimi 1983 as *Bianorpullus*)].

**Prey stage.** Larva (Kunimi 1983).

**Notes.** Collected from fall webworm nest (Kunimi 1983).

#### *Thiodina* sp.

**Recorded interactions with *H.cunea*.** USA [Arkansas (Warren et al. 1967)].

**Prey stage.** Larva (Warren et al. 1967).

**Notes.** Collected from fall webworm nest (Warren et al. 1967).

#### *Zygoballussexpunctatus* (Hentz, 1845)

**Distribution.** USA (World Spider Catalog 2023).

**Recorded interactions with *H.cunea*.** USA [Arkansas (Warren et al. 1967)].

**Prey stage.** Larva (Warren et al. 1967).

**Notes.** Collected from fall webworm nest (Warren et al. 1967).

##### ﻿﻿Tetragnathidae 长脚蛛科

#### *Tetragnathalaboriosa* Hentz, 1850

**Distribution.** Alaska to Chile, Argentina, Falkland Is. (World Spider Catalog 2023).

**Recorded interactions with *H.cunea*.** USA [Arkansas (Warren et al. 1967)].

**Prey stage.** Larva (Warren et al. 1967).

**Notes.** Collected from fall webworm nest (Warren et al. 1967).

#### *Tetragnatha* sp.

**Recorded interactions with *H.cunea*.** South of the European part of former USSR (Sharov et al. 1984).

**Prey stage.** Larva (Sharov et al. 1984).

#### *Tetragnathasquamata* Karsch, 1879

**Distribution.** Russia (Far East), China, Korea, Japan (World Spider Catalog 2023).

**Recorded interactions with *H.cunea*.** Japan [Fuchu, Akikawa (Kunimi 1983)].

**Prey stage.** Larva (Kunimi 1983).

**Notes.** Collected from fall webworm nest (Kunimi 1983).

##### ﻿﻿Theridiidae 球腹蛛科

#### *Enoplognathaovata* (Clerck, 1757)

**Distribution.** Europe, Turkey, Caucasus, Russia (Europe to Middle Siberia), Kazakhstan, Iran, Central Asia, Korea, Japan. Introduced to North America (World Spider Catalog 2023).

**Recorded interactions with *H.cunea*.** South of the European part of former USSR (Sharov et al. 1984).

**Prey stage.** Larva (Sharov et al. 1984).

#### *Euryopisfunebris* (Hentz, 1850)

**Distribution.** Canada, USA. Introduced to South Africa (World Spider Catalog 2023).

**Recorded interactions with *H.cunea*.** USA [Arkansas (Warren et al. 1967 as *Euryopsisfunebris*)].

**Prey stage.** Larva (Warren et al. 1967).

**Notes.** Collected from fall webworm nest (Warren et al. 1967).

#### *Euryopis* sp.

**Recorded interactions with *H.cunea*.** USA [Arkansas (Warren et al. 1967)].

**Prey stage.** Larva (Warren et al. 1967).

**Notes.** Collected from fall webworm nest (Warren et al. 1967).

#### *Heterotheridionnigrovariegatum* (Simon, 1873)

**Distribution.** Europe, Turkey, Caucasus, Russia (Europe) to Central Asia, Iran, China (World Spider Catalog 2023).

**Recorded interactions with *H.cunea*.** South of the European part of former USSR (Sharov et al. 1984 as *Theridionnigrovariegatum*).

**Prey stage.** Larva (Sharov et al. 1984).

#### *Neospintharustrigonum* (Hentz, 1850)

**Distribution.** USA, Canada (World Spider Catalog 2023).

**Recorded interactions with *H.cunea*.** USA [Arkansas (Warren et al. 1967 as *Argyrodestrigonum*)].

**Prey stage.** Larva (Warren et al. 1967).

**Notes.** Collected from fall webworm nest (Warren et al. 1967).

#### *Parasteatodatepidariorum* (C. L. Koch, 1841)

**Distribution.** Asia; introduced to Canada, USA, South America, Europe, Turkey, Caucasus, Russia (Europe to Far East), South Africa, Seychelles, New Zealand, Hawaii (World Spider Catalog 2023).

**Recorded interactions with *H.cunea*.** USA [Arkansas (Warren et al. 1967 as *Achaearaneatepidariorum*)], Japan (Kayashima 1967 as *Theridiontepidariorum*), Japan [Fuchu, Akikawa (Kunimi 1983 as *Achaearaneatepidariorum*)], China [Dandong City, Liaoning Province (Shu and Yu 1985 as *Theridiontepidariorum*)].

**Prey stage.** Larva (Warren et al. 1967; Kunimi 1983; Shu and Yu 1985), adult (Kayashima 1967).

**Notes.** Collected from fall webworm nest (Warren et al. 1967; Kunimi 1983).

#### *Phyllonetasisyphia* (Clerck, 1757)

**Distribution.** Europe, Turkey, Caucasus, Russia (Europe to south Siberia), Kazakhstan, Central Asia, China (World Spider Catalog 2023).

**Recorded interactions with *H.cunea*.** Italy [Po Valley (Groppali et al. 1993 as *Theridionsisyphium*)].

**Prey stage.** Larva (Groppali et al. 1993).

#### *Takayustakayensis* (Saito, 1939)

**Distribution.** China, Korea, Japan (World Spider Catalog 2023).

**Recorded interactions with *H.cunea*.** Japan [Fuchu (Kunimi 1983 as *Theridiontakayense*)].

**Prey stage.** Larva (Kunimi 1983).

**Notes.** Collected from fall webworm nest (Kunimi 1983).

#### *Teutana* sp.

**Recorded interactions with *H.cunea*.** USA [Arkansas (Warren et al. 1967)].

**Prey stage.** Larva (Warren et al. 1967).

**Notes.** Collected from fall webworm nest (Warren et al. 1967).

#### *Theridiondifferens* Emerton, 1882

**Distribution.** USA, Canada (World Spider Catalog 2023).

**Recorded interactions with *H.cunea*.** USA [Arkansas (Warren et al. 1967)].

**Prey stage.** Larva (Warren et al. 1967).

**Notes.** Collected from fall webworm nest (Warren et al. 1967).

#### *Theridionvarians* Hahn, 1833

**Distribution.** Europe, North Africa, Turkey, Caucasus, Russia (Europe to Far East), Kazakhstan, Iran, Central Asia, China. Introduced to Canada, USA (World Spider Catalog 2023).

**Recorded interactions with *H.cunea*.** South of the European part of former USSR [Moldova, Russia (Sharov et al. 1984)].

**Prey stage.** Larva (Sharov et al. 1984).

#### *Theridion* sp.

**Recorded interactions with *H.cunea*.** Japan [Akikawa (Kunimi 1983].

**Prey stage.** Larva (Kunimi 1983).

**Notes.** Collected from fall webworm nest (Kunimi 1983).

#### *Yunohamellayunohamensis* (Bösenberg & Strand, 1906)

**Distribution.** Russia (Sakhalin, Kurile Is.), Korea, Japan (World Spider Catalog 2023).

**Recorded interactions with *H.cunea*.** Japan [Akikawa (Kunimi 1983 as *Theridionyunohamense*)].

**Prey stage.** Larva (Kunimi 1983).

**Notes.** Collected from fall webworm nest (Kunimi 1983).

##### ﻿﻿Thomisidae 蟹蛛科

#### *Bassanianadecorata* (Karsch, 1879)

**Distribution.** Russia (Far East), China, Korea, Japan (World Spider Catalog 2023).

**Recorded interactions with *H.cunea*.** Japan [Fuchu, Akikawa (Kunimi 1983 as *Oxyptiladecorata*)].

**Prey stage.** Larva (Kunimi 1983).

**Notes.** Collected from fall webworm nest (Kunimi 1983).

#### *Diaeasubdola* O. Pickard-Cambridge, 1885

**Distribution.** Pakistan, India, China, Russia (Far East), Korea, Japan (World Spider Catalog 2023).

**Recorded interactions with *H.cunea*.** Japan [Fuchu, Akikawa (Kunimi 1983 as *Misumenopsjaponicus*)].

**Prey stage.** Larva (Kunimi 1983).

**Notes.** Collected from fall webworm nest (Kunimi 1983).

#### *Ebrechtellatricuspidata* (Fabricius, 1775)

**Distribution.** Europe, Turkey, Caucasus, Russia (Europe to Far East), Kazakhstan, Iran, Central Asia, China, Korea, Japan (World Spider Catalog 2023).

**Recorded interactions with *H.cunea*.** South of the European part of former USSR (Sharov et al. 1984, name as *Misumenopstricuspidatus*), Japan [Fuchu, Akikawa (Kunimi 1983 as *Misumenopstricuspidatus*)], China [Dandong City, Liaoning Province (Shu and Yu 1985 as *Misumenatricuspidatus*).

**Prey stage.** Larva (Kunimi 1983; Sharov et al. 1984; Shu and Yu 1985).

**Notes.** Collected from fall webworm nest (Kunimi 1983).

#### *Mecaphesaasperata* (Hentz, 1847)

**Distribution.** North, Central America, Caribbean (World Spider Catalog 2023).

**Recorded interactions with *H.cunea*.** USA [Arkansas (Warren et al. 1967 as *Misumenopsasperatus*)].

**Prey stage.** Larva (Warren et al. 1967).

**Notes.** Collected from fall webworm nest (Warren et al. 1967).

#### *Mecaphesaceler* (Hentz, 1847)

**Distribution.** North, Central America (World Spider Catalog 2023).

**Recorded interactions with *H.cunea*.** USA [Arkansas (Warren et al. 1967 as *Misumenopsceler*)].

**Prey stage.** Larva (Warren et al. 1967).

**Notes.** Collected from fall webworm nest (Warren et al. 1967).

#### *Misumessusoblongus* (Keyserling, 1880)

**Distribution.** Canada, USA (World Spider Catalog 2023).

**Recorded interactions with *H.cunea*.** USA [Arkansas (Warren et al. 1967 as *Misumenopsoblongus*)].

**Prey stage.** Larva (Warren et al. 1967).

**Notes.** Collected from fall webworm nest (Warren et al. 1967).

#### *Misumenops* sp.

**Recorded interactions with *H.cunea*.** USA [Arkansas (Warren et al. 1967)].

**Prey stage.** Larva (Warren et al. 1967).

**Notes.** Collected from fall webworm nest (Warren et al. 1967).

#### *Oxytatestriatipes* L. Koch, 1878

**Distribution.** Russia (Far East), China, Korea, Japan (World Spider Catalog 2023).

**Recorded interactions with *H.cunea*.** Japan [Fuchu, Akikawa (Kunimi 1983)].

**Prey stage.** Larva (Kunimi 1983; Shu and Yu 1985).

**Notes.** Collected from fall webworm nest (Kunimi 1983).

#### *Pistiustruncatus* (Pallas, 1772)

**Distribution.** Europe, Turkey, Caucasus, Russia (Europe to Far East), Iran, China (World Spider Catalog 2023).

**Recorded interactions with *H.cunea*.** Japan [Akikawa (Kunimi 1983)].

**Prey stage.** Larva (Kunimi 1983).

**Notes.** Collected from fall webworm nest (Kunimi 1983).

#### *Spiracmestriatipes* (L. Koch, 1870)

**Distribution.** Europe, Turkey, Caucasus, Russia (Europe) to Central Asia, Iran, China (World Spider Catalog 2023).

**Recorded interactions with *H.cunea*.** China [Dandong City, Liaoning Province (Shu and Yu 1985 as *Xysticusstriatipes*)].

**Prey stage.** Larva (Shu and Yu 1985).

#### *Synemaglobosum* (Fabricius, 1775)

**Distribution.** Europe, Turkey, Caucasus, Russia (Europe to Far East), Israel, Iran, Central Asia, China, Korea, Japan (World Spider Catalog 2023).

**Recorded interactions with *H.cunea*.** South of the European part of former USSR (Sharov et al. 1984).

**Prey stage.** Larva (Sharov et al. 1984).

#### *Synemaparvulum* (Hentz, 1847)

**Distribution.** USA, Mexico (World Spider Catalog 2023).

**Recorded interactions with *H.cunea*.** USA [Arkansas (Warren et al. 1967)].

**Prey stage.** Larva (Warren et al. 1967).

**Notes.** Collected from fall webworm nest (Warren et al. 1967).

#### *Tmarus* sp.

**Recorded interactions with *H.cunea*.** USA [Arkansas (Warren et al. 1967)].

**Prey stage.** Larva (Warren et al. 1967).

**Notes.** Collected from fall webworm nest (Warren et al. 1967).

#### *Thomisuslabefactus* Karsch, 1881

**Distribution.** Korea, Japan, China, Thailand (World Spider Catalog 2023).

**Recorded interactions with *H.cunea*.** Japan [Akikawa (Kunimi 1983)].

**Prey stage.** Larva (Kunimi 1983).

**Notes.** Collected from fall webworm nest (Kunimi 1983).

#### *Thomisusonustus* Walckenaer, 1805

**Distribution.** Selvagens Is. (Portugal), Europe, North Africa, Turkey, Caucasus, Russia (Europe to south Siberia), Israel, Central Asia, Iran, China, Korea, Japan (World Spider Catalog 2023).

**Recorded interactions with *H.cunea*.** South of the European part of former USSR (Sharov et al. 1984).

**Prey stage.** Larva (Sharov et al. 1984).

#### *Xysticusferox* (Hentz, 1847)

**Distribution.** USA, Canada (World Spider Catalog 2023).

**Recorded interactions with *H.cunea*.** USA [Arkansas (Warren et al. 1967)].

**Prey stage.** Larva (Warren et al. 1967).

**Notes.** Collected from fall webworm nest (Warren et al. 1967).

#### *Xysticusfunestus* Keyserling, 1880

**Distribution.** North America (World Spider Catalog 2023).

**Recorded interactions with *H.cunea*.** USA [Arkansas (Warren et al. 1967)].

**Prey stage.** Larva (Warren et al. 1967).

**Notes.** Collected from fall webworm nest (Warren et al. 1967).

#### *Xysticuslanio* C. L. Koch, 1835

**Distribution.** Europe, Turkey, Caucasus, Russia (Europe to Middle and south Siberia), Turkmenistan (World Spider Catalog 2023).

**Recorded interactions with *H.cunea*.** South of the European part of former USSR (Sharov et al. 1984).

**Prey stage.** Larva (Sharov et al. 1984).

#### *Xysticus* sp.

**Recorded interactions with *H.cunea*.** USA [Arkansas (Warren et al. 1967)].

**Prey stage.** Larva (Warren et al. 1967).

**Notes.** Collected from fall webworm nest (Warren et al. 1967).

#### *Xysticus* sp.

**Recorded interactions with *H.cunea*.** Italy [Po Valley (Groppali et al. 1993)].

**Prey stage.** Larva (Groppali et al. 1993).

##### ﻿﻿Titanoecidae 隐石蛛科

#### *Nursciaalbofasciata* (Strand, 1907)

**Distribution.** Russia (Far East), Korea, Japan, China; introduced to Britain (World Spider Catalog 2023).

**Recorded interactions with *H.cunea*.** Japan [Akikawa (Kunimi 1983)].

**Prey stage.** Larva (Kunimi 1983).

**Notes.** Collected from fall webworm nest (Kunimi 1983).

##### ﻿﻿Trachelidae 管蛛科

#### *Trachelasjaponicus* Bösenberg & Strand, 1906

**Distribution.** Russia (Kurile Is.), Korea, Japan, China (World Spider Catalog 2023).

**Recorded interactions with *H.cunea*.** Japan [Fuchu, Akikawa (Kunimi 1983)].

**Prey stage.** Larva (Kunimi 1983).

**Notes.** Collected from fall webworm nest (Kunimi 1983).

#### *Trachelassimilis* F. O. Pickard-Cambridge, 1899

**Distribution.** Canada, USA (World Spider Catalog 2023).

**Recorded interactions with *H.cunea*.** USA [Arkansas (Warren et al. 1967 as T.afflaticeps)].

**Prey stage.** Larva (Warren et al. 1967).

**Notes.** Collected from fall webworm nest (Warren et al. 1967).

#### *Trachelas* sp.

**Recorded interactions with *H.cunea*.** USA [Arkansas (Warren et al. 1967)].

**Prey stage.** Larva (Warren et al. 1967).

**Notes.** Collected from fall webworm nest (Warren et al. 1967).

#### *Trachelastranquillus* (Hentz, 1847)

**Distribution.** Canada, USA (World Spider Catalog 2023).

**Recorded interactions with *H.cunea*.** USA [Arkansas (Warren et al. 1967)].

**Prey stage.** Larva (Warren et al. 1967).

**Notes.** Collected from fall webworm nest (Warren et al. 1967).

##### ﻿﻿Uloboridae 妩蛛科

#### *Octonobavarians* (Bösenberg & Strand, 1906)

**Distribution.** China, Korea, Japan (World Spider Catalog 2023).

**Recorded interactions with *H.cunea*.** Japan [Fuchu (Kunimi 1983 as *Uloborusvarians*)].

**Prey stage.** Larva (Kunimi 1983).

**Notes.** Collected from fall webworm nest (Kunimi 1983).

##### ﻿﻿Trombidiformes

﻿**Trombidiidae** 绒螨科

#### *Allothrombidiumfuliginosum* (Hermann, 1804)

**Distribution.** Asia, Europe (CABI Compendium 2022).

**Recorded interactions with *H.cunea*.** Italy (Boriani 1991).

**Prey stage.** Egg (Boriani 1991).

##### ﻿﻿Reptilia

﻿**Squamata**

﻿**Gekkonidae** 壁虎科

#### *Geckojaponicus* (Schlegel, 1836)

**Distribution.** China, South Korea, Japan (Uetz et al. 2023).

**Recorded interactions with *H.cunea*.** Japan (Hasegawa and Itô 1967).

**Prey stage.** Adult (Hasegawa and Itô 1967).

**Notes.** Occasionally preys on *H.cunea* (Hasegawa and Itô 1967).

##### ﻿﻿Amphibia

﻿**Anura**

﻿**Bufonidae** 蟾蜍科

#### *Anaxyrusamericanus* (Holbrook, 1836)

**Distribution.** Canada, USA (Frost 2023).

**Recorded interactions with *H.cunea*.** Europe (Warren and Tadić 1967 as *Bufoamericanna*).

**Prey stage.** Larva (Warren and Tadić 1967).

#### *Bufobufo* (Linnaeus, 1758)

**Distribution.** Albania, Austria, Belarus, Belgium, Bosnia and Herzegovina, Bulgaria, China, Croatia, Czech Republic, Denmark, Estonia, Finland, France, Germany, Greece, Hungary, Italy, Jersey, Kazakhstan, Kosovo, Latvia, Liechtenstein, Lithuania, Luxembourg, Moldova, Montenegro, Netherlands, North Macedonia, Norway, Poland, Romania, Russia, San Marino, Serbia, Slovakia, Slovenia, Sweden, Switzerland, Turkey, Ukraine, United Kingdom (Frost 2023).

**Recorded interactions with *H.cunea*.** Europe (Warren and Tadić 1967).

**Prey stage.** Larva (Warren and Tadić 1967).

#### *Bufogargarizans* Cantor, 1842

**Distribution.** China, India, North Korea, South Korea, Russia, Vietnam (Frost 2023).

**Recorded interactions with *H.cunea*.** China [Dandong City, Liaoning Province (Shu and Yu 1985)].

**Prey stage.** Larva (Shu and Yu 1985).

##### ﻿﻿Ranidae 蛙科

#### *Pelophylaxnigromaculatus* (Hallowell, 1861)

**Distribution.** China, Japan, Korea, North Korea, South Korea, Mongolia, Russia (Frost 2023).

**Recorded interactions with *H.cunea*.** China [Dandong City, Liaoning Province (Shu and Yu 1985 as *Rananigromaculatus*)].

**Prey stage.** Larva (Shu and Yu 1985).

##### ﻿﻿Aves

﻿**Bucerotiformes**

﻿**Upupidae** 戴胜科

#### *Upupaepops* Linnaeus, 1758

**Distribution.** Breeds in northwestern Africa (east to northwestern Libya), Canary Islands, and central and southern Europe south to Israel, and east to southeastern Siberia and northern Korea, south to northwestern India and China; mostly migratory, winters to Africa and South Asia (Avibase 2023).

**Recorded interactions with *H.cunea*.** Italy (Camerini 1994).

**Prey stage.** Larva (Camerini 1994).

##### ﻿﻿Ciconiiformes

﻿**Ciconiidae** 鹳科

#### *Ciconiaciconia* (Linnaeus, 1758)

**Distribution.** West Palearctic and West Asia: overwinters in tropical and South Africa (Avibase 2023).

**Recorded interactions with *H.cunea*.** Europe (Warren and Tadić 1967).

**Prey stage.** Larva (Warren and Tadić 1967).

##### ﻿﻿Columbiformes

﻿**Columbidae** 鸠鸽科

#### *Streptopeliadecaocto* (Frivaldszky, 1838)

**Distribution.** Europe to Middle East, India, Sri Lanka, China, and Korea; introduced to North America and Mexico (Avibase 2023).

**Recorded interactions with *H.cunea*.** Italy (Camerini 1994).

**Prey stage.** Larva (Camerini 1994).

#### *Streptopeliaturtur* (Linnaeus, 1758)

**Distribution.** Azores, Canary Is., and Europe to West Siberia and Kazakhstan (Avibase 2023).

**Recorded interactions with *H.cunea*.** Italy (Camerini 1994).

**Prey stage.** Larva (Camerini 1994).

**Notes.** Actively prey on the *H.cunea* in September (Camerini 1994).

##### ﻿﻿Cuculiformes

﻿**Cuculidae** 杜鹃科

#### *Coccyzusamericanus* (Linnaeus, 1758)

**Distribution.** Canada to Mexico and West Indies; overwinters in Argentina (Avibase 2023).

**Recorded interactions with *H.cunea*.** North America (Warren and Tadić 1967).

**Prey stage.** Larva (Warren and Tadić 1967).

#### *Cuculuscanorus* Linnaeus, 1758

**Distribution.** Europe, Siberia to Kamchatka and Japan; overwinters in Africa (Avibase 2023).

**Recorded interactions with *H.cunea*.** North America (Warren and Tadić 1967).

**Prey stage.** Larva (Warren and Tadić 1967).

##### ﻿﻿Falconiformes

﻿**Falconidae** 隼科

#### *Falcotinnunculus* Linnaeus, 1758

**Distribution.** Europe, northwest Africa, and the Middle East to east central Siberia, Afghanistan, and western and northern Pakistan east in the Himalayas to Nepal and Bhutan; overwinters in eastern Africa and southern and southern Asia (Avibase 2023).

**Recorded interactions with *H.cunea*.** Italy (Camerini 1994).

**Prey stage.** Larva (Camerini 1994).

##### ﻿﻿Galliformes

﻿**Phasianidae** 雉科

#### *Gallusgallus* (Linnaeus, 1758)

**Distribution.** Palaearctic, Oriental (Avibase 2023).

**Recorded interactions with *H.cunea*.** Europe (Warren and Tadić 1967).

**Prey stage.** Larva (Warren and Tadić 1967).

##### ﻿﻿Passeriformes

﻿**Corvidae** 鸦科

#### *Corvuscorone* Linnaeus, 1758

**Distribution.** West Europe (Avibase 2023).

**Recorded interactions with *H.cunea*.** Europe (Warren and Tadić 1967).

**Prey stage.** Larva (Warren and Tadić 1967).

#### *Cyanopicacyanus* (Pallas, 1776)

**Distribution.** East central Asia (Avibase 2023).

**Recorded interactions with *H.cunea*.** China [Dandong City, Liaoning Province] (Shu and Yu 1985).

**Prey stage.** Larva (Shu and Yu 1985).

**Notes.** Prey heavily on *H.cunea* from early May to early June (Shu and Yu 1985).

#### *Picapica* (Linnaeus, 1758)

**Distribution.** Europe, from the British Isles, France, and southern Scandinavia to eastern Europe and Asia Minor (Avibase 2023).

**Recorded interactions with *H.cunea*.** China [Dandong City, Liaoning Province] (Shu and Yu 1985), Europe (Warren and Tadić 1967).

**Prey stage.** Larva (Shu and Yu 1985; Warren and Tadić 1967), adult (Camerini 1994).

**Notes.** Preys heavily on *H.cunea* from early May to early June (Shu and Yu 1985).

##### ﻿﻿Laniidae 伯劳科

#### *Laniuscollurio* Linnaeus, 1758

**Distribution.** Widespread in the Palearctic region, South Africa (Avibase 2023).

**Recorded interactions with *H.cunea*.** Europe (Warren and Tadić 1967).

**Prey stage.** Larva (Warren and Tadić 1967).

#### *Laniusminor* Gmelin, 1788

**Distribution.** Iberian Peninsula to Siberia and central Asia, south Africa (Avibase 2023).

**Recorded interactions with *H.cunea*.** Europe (Warren and Tadić 1967).

**Prey stage.** Larva (Warren and Tadić 1967).

##### ﻿﻿Muscicapidae 鹟科

#### *Luscinialuscinia* (Linnaeus, 1758)

**Distribution.** North Eurasia, overwinters in east and south Africa (Avibase 2023).

**Recorded interactions with *H.cunea*.** Europe (Warren and Tadić 1967).

**Prey stage.** Larva (Warren and Tadić 1967).

#### *Lusciniamegarhynchos* Brehm, 1831

**Distribution.** West Europe, North Africa, and Asia Minor; tropical Africa (Avibase 2023).

**Recorded interactions with *H.cunea*.** Italy (Camerini 1994).

**Prey stage.** Adult (Camerini 1994).

#### *Muscicapastriata* (Pallas, 1764)

**Distribution.** Europe to North Africa, Siberia, and Asia Minor (Avibase 2023).

**Recorded interactions with *H.cunea*.** Europe (Warren and Tadić 1967).

**Prey stage.** Larva (Warren and Tadić 1967).

#### *Oenantheoenanthe* (Linnaeus, 1758)

**Distribution.** British Isles to Mediterranean, and east to Siberia, Alaska, and northwest Canada (Yukon); winter to central Africa (Avibase 2023).

**Recorded interactions with *H.cunea*.** Italy (Camerini 1994).

**Prey stage.** Larva (Camerini 1994).

**Notes.** Preys on *H.cunea* during migration (Camerini 1994).

##### ﻿﻿Oriolidae 黄鹂科

#### *Oriolusoriolus* (Linnaeus, 1758)

**Distribution.** West Palearctic to east Siberia; winter to Africa and northwest India (Avibase 2023).

**Recorded interactions with *H.cunea*.** Europe (Warren and Tadić 1967).

**Prey stage.** Larva (Warren and Tadić 1967).

##### ﻿﻿Paridae 山雀科

#### *Cyanistescaeruleus* (Linnaeus, 1758)

**Distribution.** Continental Europe to north Spain, Sicily, north Turkey, and north Urals (Avibase 2023).

**Recorded interactions with *H.cunea*.** Europe (Warren and Tadić 1967).

**Prey stage.** Larva (Warren and Tadić 1967).

#### *Parusmajor* Linnaeus, 1758

**Distribution.** Palaearctic, Mediterranean, Ethiopian, Nearctic, Neotropical, Oriental (Avibase 2023).

**Recorded interactions with *H.cunea*.** China [Dandong City, Liaoning Province] (Shu and Yu 1985), Europe (Warren and Tadić 1967).

**Prey stage.** Larva (Warren and Tadić 1967), Adult (Shu and Yu 1985; Camerini 1994).

**Notes.** Actively preys on *H.cunea* larvae (Camerini 1994); preys heavily from early May to early June in Liaoning Province, China (Shu and Yu 1985); may be used as a biocontrol agent by augmenting its population using artificial broods (Camerini 1994).

##### ﻿﻿Passeridae 雀科

#### *Passerdomesticus* (Linnaeus, 1758)

**Distribution.** Palaearctic, Mediterranean, Ethiopian, Nearctic, Neotropical, Oriental, Australian (Avibase 2023).

**Recorded interactions with *H.cunea*.** China [Dandong City, Liaoning Province] (Shu and Yu 1985), Europe (Warren and Tadić 1967).

**Prey stage.** Adult (Warren and Tadić 1967), larva (Shu and Yu 1985).

**Notes.** Preys heavily from early May to early June (Shu and Yu 1985).

#### *Passermontanus* (Linnaeus, 1758)

**Distribution.** Palaearctic, Mediterranean, Ethiopian, Nearctic, Neotropical, Oriental, Australian (Avibase 2023).

**Recorded interactions with *H.cunea*.** China [Dandong City, Liaoning Province] (Shu and Yu 1985), Japan [Hiratsuka Shrine] (Hasegawa and Itô 1967). Europe (Warren and Tadić 1967).

**Prey stage.** Adult (Warren and Tadić 1967; Hasegawa and Itô 1967; Camerini 1994), larva (Shu and Yu 1985).

**Notes.** Preys heavily from early May to early June (Shu and Yu 1985), played an important role in suppressing the reproduction of *H.cunea* (Hasegawa and Itô 1967).

##### ﻿﻿Sturnidae 椋鸟科

#### *Sturnusvulgaris* Linnaeus, 1758

**Distribution.** Canary Is. and Iceland to Ural Mts., north Ukraine, and southeast Europe (Avibase 2023).

**Recorded interactions with *H.cunea*.** Italy (Camerini 1994).

**Prey stage.** Larva (Warren and Tadić 1967), pupa and adult (Camerini 1994).

#### *Spodiopsarcineraceus* (Temminck, 1835)

**Distribution.** Northeast Asia; winters in south China and Philippines (Avibase 2023).

**Recorded interactions with *H.cunea*.** Japan [Hiratsuka Shrine] (Hasegawa and Itô 1967).

**Prey stage.** Adult (Hasegawa and Itô 1967; Camerini 1994).

**Notes.** Played an important role in suppressing the reproduction of *H.cunea* (Hasegawa and Itô 1967).

##### ﻿﻿Sylviidae 莺科

#### *Currucacurruca* (Linnaeus, 1758)

**Distribution.** East Siberia to north Altai and north Mongolia (Avibase 2023).

**Recorded interactions with *H.cunea*.** Europe (Warren and Tadić 1967).

**Prey stage.** Larva (Warren and Tadić 1967).

##### ﻿﻿Turdidae 鸫科

#### *Saxicolarubetra* (Linnaeus, 1758)

**Distribution.** West Palearctic; tropical and south Africa (Avibase 2023).

**Recorded interactions with *H.cunea*.** Italy (Camerini 1994).

**Prey stage.** Larva (Camerini 1994).

**Notes.** Preys on *H.cunea* during migrations (Camerini 1994).

#### *Turdusmerula* Linnaeus, 1758

**Distribution.** West Europe; introduced Southeast Australia, Tasmania, Norfolk, Lord Howe Is. (Avibase 2023).

**Recorded interactions with *H.cunea*.** Europe (Warren and Tadić 1967).

**Prey stage.** Larva (Warren and Tadić 1967).

#### *Turdusphilomelos* Brehm, 1831

**Distribution.** North and east Europe to central Asia; winters to North Africa and Iran (Avibase 2023).

**Recorded interactions with *H.cunea*.** Europe (Warren and Tadić 1967).

**Prey stage.** Larva (Warren and Tadić 1967).

##### ﻿﻿Vireonidae 绿鹃科

#### *Vireoolivaceus* (Linnaeus, 1766)

**Distribution.** West-central and Eastern US; overwinters in Cuba and central South America (Avibase 2023).

**Recorded interactions with *H.cunea*.** North America (Warren and Tadić 1967).

**Prey stage.** Larva (Warren and Tadić 1967).

##### ﻿﻿Piciformes

﻿**Picidae** 啄木鸟科

#### *Dendrocoposmajor* (Linnaeus, 1758)

**Distribution.** Palaearctic, Mediterranean, Oriental (Avibase 2023).

**Recorded interactions with *H.cunea*.** Italy (Camerini 1994).

**Prey stage.** Larva (Warren and Tadić 1967), pupa (Camerini 1994).

#### *Dendrocoposmedius* (Linnaeus, 1758)

**Distribution.** Northwest Spain to France, Estonia, West Russia, Ukraine, Italy, Balkans (Avibase 2023).

**Recorded interactions with *H.cunea*.** Europe (Warren and Tadić 1967).

**Prey stage.** Larva (Warren and Tadić 1967).

#### *Dendrocopossyriacus* (Hemprich & Ehrenberg, 1833)

**Distribution.** Southeast Europe, Transcaucasia, Turkey, and Iran to Israel and Jordan (Avibase 2023).

**Recorded interactions with *H.cunea*.** Europe (Warren and Tadić 1967).

**Prey stage.** Larva (Warren and Tadić 1967).

##### ﻿﻿Strigiformes

﻿**Strigidae** 鸱鸮科

#### *Asiootus* (Linnaeus, 1758)

**Distribution.** Europe, Asia, North Africa, North America (Avibase 2023).

**Recorded interactions with *H.cunea*.** North America (Warren and Tadić 1967).

**Prey stage.** Larva (Warren and Tadić 1967).

### ﻿﻿Part II Parasitoids

﻿**Hymenoptera**

﻿**Ichneumonidae** 姬蜂科

#### *Apechthiscompunctor* (Linnaeus, 1758)

**Distribution.** Asia, Europe, USA (Yu et al. 2016).

**Recorded interactions with *H.cunea*.** Former USSR (Yu et al. 2016).

**Host stage.** Prepupa (Yu et al. 2016).

**Parasitoid type.** Endoparasitoid, solitary, koinobiont (Yu et al. 2016).

#### *Casinariagenuina* (Norton, 1863)

**Distribution.** Canada, USA (Yu et al. 2016).

**Recorded interactions with *H.cunea*.** USA [New England **or** New York **or** New Jersey (Schaffner and Griswold 1934 as *Neonortoniamajor*); Colorado (Swain 1937 as *Neonortoniamajor*)].

**Host stage.** Larva (Warren and Tadić 1967).

**Parasitoid type.** Endoparasitoid (Yu et al. 2016).

#### *Casinarialimenitidis* (Howard, 1889)

**Distribution.** Canada, USA (Yu et al. 2016).

**Recorded interactions with *H.cunea*.** USA [New England **or** New York **or** New Jersey (Schaffner and Griswold 1934 as *C.orgyiae*); Colorado (Swain 1937 as *C.orgyiae*)].

**Host stage.** Larva (Warren and Tadić 1967).

**Parasitoid type.** Endoparasitoid (Yu et al. 2016).

#### *Casinariaischnogaster* Thomson, 1887

**Distribution.** Europe, Palaearctic (Yu et al. 2016).

**Recorded interactions with *H.cunea*.** Former USSR (Yu et al. 2016).

**Host stage.** Larva (Yu et al. 2016).

**Parasitoid type.** Endoparasitoid, solitary (Yu et al. 2016).

#### *Casinarianigripes* (Gravenhorst, 1829)

**Distribution.** Europe, Palaearctic, Oriental (Yu et al. 2016).

**Recorded interactions with *H.cunea*.** China [Dandong City, Liaoning Province (Shu and Yu 1985 as *Apantelesardimarium*)].

**Host stage.** Larva (Shu and Yu 1985).

**Parasitoid type.** Endoparasitoid, solitary (Shu and Yu 1985).

#### *Cratichneumonculex* (Müller, 1776)

**Distribution.** Europe, Palaearctic (Yu et al. 2016).

**Recorded interactions with *H.cunea*.** Former USSR (Yu et al. 2016).

**Host stage.** Larva, pupa (Yu et al. 2016).

**Parasitoid type.** Endoparasitoid, solitary, polyphagous (Yu et al. 2016).

#### *Cratichneumonluteiventris* (Gravenhorst, 1820)

**Distribution.** Europe, Palaearctic (Yu et al. 2016).

**Recorded interactions with *H.cunea*.** Former USSR (Yu et al. 2016).

**Host stage.** Larva, emerge from pupa (Yu et al. 2016).

**Parasitoid type.** Endoparasitoid, polyphagous (Yu et al. 2016).

#### *Diradopshyphantriae* Kasparyan & Pinson, 2007

**Distribution.** Mexico (Yu et al. 2016).

**Recorded interactions with *H.cunea*.** Mexico (Kasparyan and Pinson 2007).

**Host stage.** Larva (Kasparyan and Pinson 2007).

**Parasitoid type.** Koinobiont endoparasitoid (Kasparyan and Pinson 2007).

#### *Enicospiluslineolatus* (Roman, 1913)

**Distribution.** Brunei, China, India, Indonesia, Japan, Korea, Malaysia, Nepal, New Caledonia, Papua New Guinea, Philippines, Sri Lanka (Yu et al. 2016).

**Recorded interactions with *H.cunea*.** China (Yang et al. 2008).

**Host stage.** Larva-prepupa (Yang et al. 2008).

**Parasitoid type.** Solitary (Yang et al. 2008).

**Notes.** Parasitoid female to male ratio 1.8 (Yang et al. 2008); parasitism rate 1.5% (Yang et al. 2008).

#### *Enicospilusramidulus* (Linnaeus, 1758)

**Distribution.** Europe, Asia (Yu et al. 2016).

**Recorded interactions with *H.cunea*.** Turkey [Samsun Province (Sullivan et al. 2010)].

**Host stage.** Pupa (Sullivan et al. 2010).

**Parasitoid type.** Endoparasitoid, solitary (Sullivan et al. 2010), polyphagous (Yu et al. 2016).

#### *Enicospilusglabratus* (Say, 1835)

**Distribution.** North America, South America (Yu et al. 2016).

**Recorded interactions with *H.cunea*.** USA [Colorado (Swain 1937 as *Eremotylusglabratus*)].

**Host stage.** Larva (Warren and Tadić 1967).

**Parasitoid type.** Polyphagous (Yu et al. 2016).

#### *Gelis* sp.

**Recorded interactions with *H.cunea*.** Turkey [Samsun Province (Sullivan et al. 2010)].

**Host stage.** Pupa (Sullivan et al. 2010).

**Parasitoid type.** Endoparasitoid, solitary (Sullivan et al. 2010), polyphagous (Yu et al. 2016).

#### *Gotraoctocincta* (Ashmead, 1906)

**Distribution.** China, Japan, Korea (Yu et al. 2016).

**Recorded interactions with *H.cunea*.** China [Binzhou City, Shandong Province (Li 2011)].

**Host stage.** Larva-pupa (Li 2011).

**Parasitoid type.** Polyphagous (Yu et al. 2016).

**Notes.** Parasitism rate 0.2% (Li 2011).

#### *Gregopimplainquisitor* (Scopoli, 1763)

**Distribution.** Palaearctic, Europe, Canada (Yu et al. 2016).

**Recorded interactions with *H.cunea*.** Former USSR, South Korea (Yu et al. 2016).

**Host stage.** Larva, emerge from pupa (Yu et al. 2016).

**Parasitoid type.** Endoparasitoid, polyphagous (Yu et al. 2016).

#### *Gregopimplakuwanae* (Viereck, 1912)

**Distribution.** Palaearctic, Oriental (Yu et al. 2016).

**Recorded interactions with *H.cunea*.** China [Tai’an City, Shandong Province (Li 2011)].

**Host stage.** Larva-pupa (Li 2011).

**Parasitoid type.** Polyphagous (Yu et al. 2016).

**Notes.** Parasitism rate 1.3% (Li 2011).

#### *Gregopimplamalacosomae* (Seyrig, 1927)

**Distribution.** Palaearctic, Europe (Yu et al. 2016).

**Recorded interactions with *H.cunea*.** Europe (Sullivan et al. 2010).

**Host stage.** Larva (Sullivan et al. 2010).

**Parasitoid type.** Polyphagous (Yu et al. 2016).

#### *Heterischnustruncator* (Fabricius, 1798)

**Distribution.** Palaearctic, Europe (Yu et al. 2016).

**Recorded interactions with *H.cunea*.** Former USSR (Yu et al. 2016).

**Host stage.** Larva, emerged from pupa (Yu et al. 2016).

**Parasitoid type.** Endoparasitoid, polyphagous (Yu et al. 2016).

#### *Hyposoterfugitivus* (Say, 1835)

**Distribution.** Brazil, Canada, USA (Yu et al. 2016).

**Recorded interactions with *H.cunea*.** USA [Colorado (Swain 1937)], Canada (Morris 1976b).

**Host stage.** Early instar larvae (Morris 1976b).

**Parasitoid type.** Solitary, endoparasitoid, koinobiont (Morris 1976b), polyphagous (Yu et al. 2016).

**Notes.** Parasitoid female to male ratio 0.67 (Morris 1976b).

#### *Hyposoterrivalis* (Cresson, 1872)

**Distribution.** China, Canada, USA (Yu et al. 2016).

**Recorded interactions with *H.cunea*.** USA [Colorado (Swain 1937 as *H.pilosulus*)], Canada (Morris 1976b as *H.pilosulus*).

**Host stage.** Early instar larvae (Morris 1976b).

**Parasitoid type.** Solitary, endoparasitoid, koinobiont (Morris 1976b), polyphagous (Yu et al. 2016).

**Notes.** Parasitoid female to male ratio 1.27 (Morris 1976b).

#### *Ichneumondeliratorius* Linnaeus, 1758

**Distribution.** Europe, Palaearctic, Nearctic (Yu et al. 2016).

**Recorded interactions with *H.cunea*.** North America (Townes 1944 as *Pterocormuscinctitarsis*).

**Host stage.** Larva (Warren and Tadić 1967).

**Parasitoid type.** Endoparasitoid, polyphagous (Yu et al. 2016).

#### *Iseropusstercorator* (Fabricius, 1793)

**Distribution.** Europe, Palaearctic, Nearctic (Yu et al. 2016).

**Recorded interactions with *H.cunea*.** Former USSR (Yu et al. 2016).

**Host stage.** Larva, emerged from pupa (Yu et al. 2016).

**Parasitoid type.** Endoparasitoid, polyphagous (Yu et al. 2016).

#### *Itoplectisalternans* (Gravenhorst, 1829)

**Distribution.** Europe, Palaearctic, USA (Yu et al. 2016).

**Recorded interactions with *H.cunea*.** Former USSR (Yu et al. 2016).

**Host stage.** Larva (Yu et al. 2016).

**Parasitoid type.** Polyphagous (Yu et al. 2016).

#### *Itoplectisconquisitor* (Say, 1835)

**Distribution.** Nearctic, Neotropical, Oceanic (Yu et al. 2016).

**Recorded interactions with *H.cunea*.** USA [Colorado (Swain 1937)], North America (Townes 1944).

**Host stage.** Larva (Warren and Tadić 1967).

**Parasitoid type.** Polyphagous (Yu et al. 2016).

#### *Itoplectismaculator* (Fabricius, 1775)

**Distribution.** Europe, Palaearctic, Oriental (Yu et al. 2016).

**Recorded interactions with *H.cunea*.** Europe (Sullivan et al. 2010).

**Host stage.** Larva, emerged from pupa (Sullivan et al. 2010).

**Parasitoid type.** Polyphagous (Yu et al. 2016).

#### *Itoplectisviduata* (Gravenhorst, 1829)

**Distribution.** Europe, Palaearctic, Nearctic (Yu et al. 2016).

**Recorded interactions with *H.cunea*.** Europe (Sullivan et al. 2010).

**Host stage.** Larva, emerged from pupa (Sullivan et al. 2010).

**Parasitoid type.** Polyphagous (Yu et al. 2016).

#### Netelia (Netelia) testacea (Gravenhorst, 1829)

**Distribution.** Afrotropical, Australasian, Europe, Neotropical, Palaearctic, Oceanic, Oriental (Yu et al. 2016).

**Recorded interactions with *H.cunea*.** Europe (Warren and Tadić 1967).

**Host stage.** Pupa (Warren and Tadić 1967).

**Parasitoid type.** Polyphagous (Yu et al. 2016).

#### *Phobocampepallipes* (Provancher, 1875)

**Distribution.** Canada, USA (Yu et al. 2016).

**Recorded interactions with *H.cunea*.** USA [Colorado (Swain 1937 as *Hyposoterpallipes*)].

**Host stage.** Larva (Warren and Tadić 1967).

**Parasitoid type.** Polyphagous (Yu et al. 2016).

#### *Phygadeuonvariabilis* Gravenhorst, 1829

**Distribution.** Europe, Palaearctic, Oriental (Yu et al. 2016).

**Recorded interactions with *H.cunea*.** Former USSR (Yu et al. 2016).

**Host stage.** Larva, emerged from pupa (Yu et al. 2016).

**Parasitoid type.** Endoparasitoid, polyphagous (Yu et al. 2016).

#### *Pimplaaethiops* Curtis, 1828

**Distribution.** Europe, East Asia (Yu et al. 2016).

**Recorded interactions with *H.cunea*.** China (Yang et al. 2008 as *Coccygomimusparnarae*).

**Host stage.** Pupa (Yang et al. 2008).

**Parasitoid type.** Solitary (Yang et al. 2008).

**Notes.** Parasitoid female to male ratio 2 (Yang et al. 2008); parasitism rate 0.34% (Yang et al. 2008).

#### *Pimplaaterrima* Gravenhorst, 1829

**Distribution.** Europe, Palaearctic (Yu et al. 2016).

**Recorded interactions with *H.cunea*.** Asia (Warren and Tadić 1967).

**Host stage.** Pupa (Warren and Tadić 1967).

**Parasitoid type.** Polyphagous (Yu et al. 2016).

#### *Pimpladisparis* Viereck, 1911

**Distribution.** East Asia, USA (Yu et al. 2016).

**Recorded interactions with *H.cunea*.** Japan [Tokyo (Tamura 1969 as *Coccygomimusdisparis*)], China [Dandong City, Liaoning Province (Shu and Yu 1985 as *Coccygomimusdisparis*)], Wugong Conty, Shaanxi Province (Ran and Zhao 1989 as *Coccygomimusdisparis*)], China (Yang et al. 2008 as *Coccygomimusdisparis*).

**Host stage.** Pupa (Ran and Zhao 1989; Yang et al. 2008).

**Parasitoid type.** Solitary (Yang et al. 2008), koinobiont (Ran and Zhao 1989).

**Notes.** Parasitoid female to male ratio 2.78 (Yang et al. 2008); parasitism rate 10% (Yang et al. 2008).

#### *Pimplaluctuosa* Smith, 1874

**Distribution.** East Asia (Yu et al. 2016).

**Recorded interactions with *H.cunea*.** China [Wugong Conty, Shaanxi Province (Ran and Zhao 1989 as *Coccygomimusluctuosus*), Dandong City, Liaoning Province (Shu and Yu 1985 as *Coccygomimusluctuosus*)].

**Host stage.** Pupa (Ran and Zhao 1989), mature larva and pupa (Shu and Yu 1985).

**Parasitoid type.** Solitary, koinobiont (Ran and Zhao 1989).

**Notes.** Parasitism rate 4–6% (Shu and Yu 1985).

#### *Pimplapedalis* Cresson, 1865

**Distribution.** Canada, USA (Yu et al. 2016).

**Recorded interactions with *H.cunea*.** North America (Warren and Tadić 1967).

**Host stage.** Larva (Warren and Tadić 1967).

**Parasitoid type.** Polyphagous (Yu et al. 2016).

#### *Pimplarufipes* (Miller, 1759)

**Distribution.** Europe, Asia, Oceanic (Yu et al. 2016).

**Recorded interactions with *H.cunea*.** Europe (Warren and Tadić 1967 as *P.instigator*), Turkey [Samsun Province (Sullivan et al. 2010)], Iran [Guilan province (Karami et al. 2023)].

**Host stage.** Pupa (Sullivan et al. 2010).

**Parasitoid type.** Endoparasitoid, solitary (Sullivan et al. 2010), polyphagous (Yu et al. 2016).

#### *Pimplaspuria* Gravenhorst, 1829

**Distribution.** Europe, Asia (Yu et al. 2016).

**Recorded interactions with *H.cunea*.** Europe (Warren and Tadić 1967).

**Host stage.** Pupa (Warren and Tadić 1967).

**Parasitoid type.** Polyphagous (Yu et al. 2016).

#### *Pimplaturionellae* (Linnaeus, 1758)

**Distribution.** Europe, Nearctic, Asia (Yu et al. 2016).

**Recorded interactions with *H.cunea*.** China (Yang et al. 2008 as *Coccygomimusturionellae*).

**Host stage.** Pupa (Yang et al. 2008).

**Parasitoid type.** Solitary parasitism (Yang et al. 2008).

**Notes.** Parasitoid female to male ratio 1.6 (Yang et al. 2008); parasitism rate 0.12% (Yang et al. 2008).

#### Rhimphoctona (Xylophylax) megacephalus (Gravenhorst, 1829)

**Distribution.** Europe, Palaearctic, Oriental (Yu et al. 2016).

**Recorded interactions with *H.cunea*.** Europe (Warren and Tadić 1967 as *Pyracmonaustriacus*).

**Host stage.** Pupa (Warren and Tadić 1967).

**Parasitoid type.** Polyphagous (Yu et al. 2016).

#### *Sinophorusvalidus* (Cresson, 1864)

**Distribution.** Canada, USA (Yu et al. 2016).

**Recorded interactions with *H.cunea*.** USA [Colorado (Swain 1937 as *Eulimneriavalida*)], Canada (Morris 1976b).

**Host stage.** First instar larvae (Morris 1976b).

**Parasitoid type.** Solitary parasitism, endoparasitoid, koinobiont (Morris 1976b), polyphagous (Yu et al. 2016).

**Notes.** Adults of *S.validus* emerge a week or two later than those of *H.cunea* and attack first-instar larvae (Morris 1976b). Parasitoid female to male ratio 0.92 (Morris 1976b).

#### *Therionmorio* (Fabricius, 1781)

**Distribution.** Canada, Mexico, Panama, USA (Yu et al. 2016).

**Recorded interactions with *H.cunea*.** USA [Colorado (Swain 1937)], North America (Warren and Tadić 1967).

**Host stage.** Pupa (Warren and Tadić 1967).

**Parasitoid type.** Polyphagous (Yu et al. 2016).

#### *Therionsassacus* Viereck, 1917

**Distribution.** Canada, Mexico, USA (Yu et al. 2016).

**Recorded interactions with *H.cunea*.** USA [Colorado (Swain 1937)], Canada (Morris 1976b).

**Host stage.** Larva of late instar (Morris 1976b).

**Parasitoid type.** Solitary, endoparasitoid, koinobiont (Morris 1976b), polyphagous (Yu et al. 2016).

**Notes.** Parasitoid female to male ratio 0.89 (Morris 1976b).

#### *Theroniaatalantae* (Poda, 1761)

**Distribution.** Europe, Palaearctic, Oriental, USA (Yu et al. 2016).

**Recorded interactions with *H.cunea*.** Europe (Warren and Tadić 1967).

**Host stage.** Pupa (Warren and Tadić 1967).

**Parasitoid type.** Polyphagous (Yu et al. 2016).

#### *Theroscopusesenbeckii* (Gravenhorst, 1815)

**Distribution.** Europe, West Palaearctic (Yu et al. 2016).

**Recorded interactions with *H.cunea*.** Europe (Warren and Tadić 1967 as *Hemitelesinaequalis* and *H.subzonatus*).

**Host stage.** Pupa (Warren and Tadić 1967).

**Parasitoid type.** Polyphagous (Yu et al. 2016).

#### *Trychosislegator* (Thunberg, 1822)

**Distribution.** Palaearctic (Yu et al. 2016).

**Recorded interactions with *H.cunea*.** Europe (Warren and Tadić 1967 as *T.ingratus*).

**Host stage.** Pupa (Warren and Tadić 1967).

**Parasitoid type.** Polyphagous (Yu et al. 2016).

#### *Virgichneumonalbilineatus* (Gravenhorst, 1820)

**Distribution.** Europe, Asia (Yu et al. 2016).

**Recorded interactions with *H.cunea*.** Turkey [Samsun Province (Sullivan et al. 2010)].

**Host stage.** Pupa (Sullivan et al. 2010).

**Parasitoid type.** Endoparasitoid, solitary (Sullivan et al. 2010), polyphagous (Yu et al. 2016).

#### *Virgichneumondumeticola* (Gravenhorst, 1829)

**Distribution.** Europe, Asia (Yu et al. 2016).

**Recorded interactions with *H.cunea*.** Turkey [Samsun Province (Sullivan et al. 2010)], Iran [Guilan province (Karami et al. 2023)].

**Host stage.** Pupa (Sullivan et al. 2010).

**Parasitoid type.** Endoparasitoid, solitary (Sullivan et al. 2010), polyphagous (Yu et al. 2016).

**Notes.** Parasitoid female to male ratio 2.16 (Sullivan et al. 2010).

#### *Virgichneumonsubcyaneus* (Cresson, 1864)

**Distribution.** Canada, USA (Yu et al. 2016).

**Recorded interactions with *H.cunea*.** USA [New England **or** New York **or** New Jersey (Schaffner and Griswold 1934 as *Amblytelespullatus*), Colorado (Swain 1937 as *Amblytelessubcyaneus*)], North America (Warren and Tadić 1967 as *Vulgichneumonsubcyaneus*).

**Host stage.** Larva (Warren and Tadić 1967).

**Parasitoid type.** Polyphagous (Yu et al. 2016).

#### *Vulgichneumonbrevicinctor* (Say, 1825)

**Distribution.** Canada, USA (Yu et al. 2016).

**Recorded interactions with *H.cunea*.** USA [New England **or** New York **or** New Jersey (Schaffner and Griswold 1934 as *Amblytelesbrevicinctor*), Colorado (Swain 1937 as *Amblytelesbrevicinctor*)].

**Host stage.** Larva (Warren and Tadić 1967).

**Parasitoid type.** Solitary, polyphagous (Yu et al. 2016).

#### *Vulgichneumonleucaniae* (Uchida, 1924)

**Distribution.** China, Japan, Russa (Yu et al. 2016).

**Recorded interactions with *H.cunea*.** China [Dandong City, Liaoning Province (Shu and Yu 1985); Wugong Conty, Shaanxi Province (Ran and Zhao 1989)].

**Host stage.** Pupa (Ran and Zhao 1989).

**Parasitoid type.** Solitary, koinobiont (Ran and Zhao 1989).

##### ﻿﻿Braconidae 茧蜂科

#### *Aleiodessanctihyacinthi* (Provancher, 1880)

**Distribution.** Canada, USA, Serbia (Yu et al. 2016).

**Recorded interactions with *H.cunea*.** USA [Colorado (Swain 1937 as *Rogashyphantriae*)].

**Host stage.** Larva (Warren and Tadić 1967).

**Parasitoid type.** Polyphagous (Yu et al. 2016).

#### Apanteles (Dolichogenidea) lacteicolor Viereck, 1911

**Distribution.** Europe, Asia, North America (Yu et al. 2016).

**Recorded interactions with *H.cunea*.** USA [Colorado (Swain 1937 as *Apanteleslacteicolor*), New English (Marsh 1979)].

**Host stage.** Larva (Warren and Tadić 1967).

**Parasitoid type.** Polyphagous (Yu et al. 2016).

#### Apanteles (Pholetesor) glacialis (Ashmead, 1902)

**Distribution.** North America (Yu et al. 2016).

**Recorded interactions with *H.cunea*.** USA [Alaska (Marsh 1979)].

#### *Apantelessingularis* (Yang & You, 2002)

**Distribution.** China (Yu et al. 2016).

**Recorded interactions with *H.cunea*.** China [Yangling City, Shaanxi Province; Yantai City, Shandong Province (Yang et al. 2002 as *Dolichogenideasingularis*)].

**Host stage.** Larva 1–3 instar (Yang et al. 2002).

**Parasitoid type.** Koinobiont, primary, solitary, endoparasitoid, polyphagous (Yang et al. 2002).

**Notes.** 5–6% parasitism (Yang et al. 2002).

#### *Cotesiadiacrisiae* (Gahan, 1917)

**Distribution.** Canada, USA (Yu et al. 2016).

**Recorded interactions with *H.cunea*.** USA [Colorado (Swain 1937 as *Apantelesdiacrisiae*)].

**Host stage.** Larva (Warren and Tadić 1967).

**Parasitoid type.** Polyphagous (Yu et al. 2016).

#### *Cotesiagregalis* Yang & Wei, 2002

**Distribution.** China (Yu et al. 2016).

**Recorded interactions with *H.cunea*.** China [Tianjin City; Qinhuangdao City, Hebei Province; Dalian City, Liaoning Province; Yantai City, Shandong Province (Yang et al. 2002)].

**Host stage.** Larva (Yang et al. 2002).

**Parasitoid type.** Koinobiont, primary, gregarious, endoparasitoid (Yang et al. 2002).

**Notes.** Parasitism rate 6% (Yang et al. 2002).

#### *Cotesiahyphantriae* (Riley, 1887)

**Distribution.** Bulgaria, Canada, China, Czech, Czechoslovakia, Germany, Greece, Hungary, Iran, Japan, Korea, Mexico, Moldova, Netherlands, Poland, Romania, Russia, Slovakia, Switzerland, Turkey, USA, Ukraine, UK, Yugoslavia (Yu et al. 2016).

**Recorded interactions with *H.cunea*.** USA [Colorado (Swain 1937 as *Apanteleshyphantriae*)], Turkey [Düzce (Avci et al. 2022 as *Apanteleshyphantriae*)]. Canada (Morris 1976b as *Apanteleshyphantriae*).

**Host stage.** Larva (Morris 1976b), egg and larva (Warren and Tadić 1967).

**Parasitoid type.** Solitary, endoparasitoid, koinobiont (Morris 1976b), polyphagous parasitism (Yu et al. 2016).

**Notes.** Parasitoid female to male ratio 1.38 (Morris 1976b).

#### *Cotesiaordinaria* (Ratzeburg, 1844)

**Distribution.** Asia, Europe (Yu et al. 2016).

**Recorded interactions with *H.cunea*.** China [Dandong City, Liaoning Province (Shu and Yu 1985 as *Apantelesardimarium*)].

**Host stage.** Larva (Shu and Yu 1985).

**Parasitoid type.** Endoparasitoid, gregarious parasitism (Shu and Yu 1985).

#### *Cotesiaplutellae* (Kurdjumov, 1912)

**Distribution.** Widespread all over the world (Yu et al. 2016).

**Recorded interactions with *H.cunea*.** Hungry [Hédervár (Papp 1988 as *Apantelesplutellae*)], Europe (Warren and Tadić 1967 as *Apantelesplutellae*).

**Host stage.** Larva (Warren and Tadić 1967).

**Parasitoid type.** Polyphagous (Yu et al. 2016).

#### *Cotesiaruficrus* (Haliday, 1834)

**Distribution.** Widespread all over the world (Yu et al. 2016).

**Recorded interactions with *H.cunea*.** Hungry [Újszentmargita (Papp 1988 as *Apantelesruficrus*)], Europe (Warren and Tadić 1967 as *Apantelesruficrus*).

**Host stage.** Larva (Warren and Tadić 1967).

**Parasitoid type.** Polyphagous (Yu et al. 2016).

#### *Cotesiavanessae* (Reinhard, 1880)

**Distribution.** Asia, North Africa, North America, Europe (Yu et al. 2016).

**Recorded interactions with *H.cunea*.** Europe (Warren and Tadić 1967 as *Apantelesvanessae*).

**Host stage.** Larva (Warren and Tadić 1967).

**Parasitoid type.** Polyphagous (Yu et al. 2016).

#### *Meteorusacronyctae* Muesebeck, 1923

**Distribution.** USA (Yu et al. 2016).

**Recorded interactions with *H.cunea*.** USA [Colorado (Swain 1937)].

**Host stage.** Larva (Warren and Tadić 1967).

**Parasitoid type.** Polyphagous (Yu et al. 2016).

#### *Meteorusbakeri* Cook & Davis, 1891

**Distribution.** Canada, USA (Yu et al. 2016).

**Recorded interactions with *H.cunea*.** USA [Colorado (Swain 1937)].

**Host stage.** Larva (Warren and Tadić 1967).

**Parasitoid type.** Polyphagous (Yu et al. 2016).

#### *Meteorushyphantriae* Riley, 1887

**Distribution.** Canada, USA (Yu et al. 2016).

**Recorded interactions with *H.cunea*.** USA [Washington DC (Riley 1887b), Colorado (Swain 1937)].

**Host stage.** Larva (Riley 1887b; Warren and Tadić 1967).

**Parasitoid type.** Polyphagous (Yu et al. 2016).

**Notes.** Performed well as natural enemy during outbreak and prevented further increase in *H.cunea* populations (Riley 1887b). The parasitism rate 0.08–3.94% of black fall webworm race, and 0.24–5.71% of orange race (Oliver 1964).

#### *Meteoruspendulus* (Müller, 1776)

**Distribution.** Asia, North America, Europe (Yu et al. 2016).

**Recorded interactions with *H.cunea*.** USA [Colorado (Swain 1937 as *M.communis*)].

**Host stage.** Larva (Warren and Tadić 1967 as *M.communis*).

**Parasitoid type.** Polyphagous (Yu et al. 2016).

#### *Meteorusversicolor* (Wesmael, 1835)

**Distribution.** Canada, USA (Yu et al. 2016).

**Recorded interactions with *H.cunea*.** USA [Colorado (Swain 1937)], Canada [New Brunswick and Nova Scotia (Morris 1976c)].

**Host stage.** Larva (Warren and Tadić 1967).

**Parasitoid type.** Polyphagous (Yu et al. 2016).

#### *Microplitishyphantriae* Ashmead, 1898

**Distribution.** Canada, USA (Yu et al. 2016).

**Recorded interactions with *H.cunea*.** USA [Washington DC (Ashmead 1898)].

**Host stage.** Larva (Warren and Tadić 1967).

**Parasitoid type.** Polyphagous (Yu et al. 2016).

##### ﻿﻿Trigonalidae 钩腹蜂科

#### *Lycogasterpullatanevadensis* (Cresson, 1879)

**Distribution.** Nearctic (Townes 1956).

**Recorded interactions with *H.cunea*.** USA [Boulder, Colorado (Townes 1956)], North America (Warren and Tadić 1967).

**Host stage.** Larva (Warren and Tadić 1967).

**Parasitoid type.** Probably a hyperparasitoid (Townes 1956).

##### ﻿﻿Chalcididae 小蜂科

#### *Brachymeriafemorata* (Panzer, 1801)

**Distribution.** Europe, Asia (Noyes 2019).

**Recorded interactions with *H.cunea*.** Europe (Warren and Tadić 1967).

**Host stage.** Pupa (Warren and Tadić 1967).

**Parasitoid type.** Endoparasitoid, polyphagous (Noyes 2019).

#### *Brachymerialasus* (Walker, 1841)

**Distribution.** Australian, Europe, Asia, North Africa, USA (Noyes 2019).

**Recorded interactions with *H.cunea*.** Japan [Tokyo (Tamura 1969 as *B.obscurata*), China [Wugong Conty, Shaanxi Province (Ran and Zhao 1989)], China (Yang et al. 2008), Iran [Guilan province (Karami et al. 2023)].

**Host stage.** Larva (Ran and Zhao 1989), Pupa only in summer generations (Yang et al. 2008).

**Parasitoid type.** Solitary, polyphagous (Ran and Zhao 1989), gregarious parasitism (Yang et al. 2008).

**Notes.** Parasitoid female to male ratio 6.4 (Yang et al. 2008); parasitism rate 6.6–16.7% (Yang et al. 2008).

#### *Brachymeriaovata* (Say, 1824)

**Distribution.** Nearctic, Neotropical (Noyes 2019).

**Recorded interactions with *H.cunea*.** North America (Peck 1963).

**Host stage.** Pupa (McDermott 1911; Burks 1936).

**Parasitoid type.** Polyphagous (Noyes 2019).

#### *Brachymeriatibialis* (Walker, 1834)

**Distribution.** Europe, North Africa, East Asia, South Asia, USA (Noyes 2019).

**Recorded interactions with *H.cunea*.** Europe (Warren and Tadić 1967 as *B.intermedia*), Italy (Boriani 1991 as *B.intermedia*).

**Host stage.** Pupa (Warren and Tadić 1967).

**Parasitoid type.** Endoparasitoid, polyphagous (Noyes 2019).

#### *Brachymeriasubconica* Boucek, 1992

**Distribution.** Nearctic, Neotropical (Noyes 2019).

**Recorded interactions with *H.cunea*.** Mexico [Nuevo Leon (Noyes 2019)].

**Host stage.** Larva (Noyes 2019).

**Parasitoid type.** Primary, polyphagous (Noyes 2019).

#### *Conurameteori* Burks, 1940

**Distribution.** Canada, USA, Mexico (Noyes 2019).

**Recorded interactions with *H.cunea*.** North America (Peck 1963 as *Ceratostnicrameteori*).

**Host stage.** Cocoon (Riley 1888).

**Parasitoid type.** Hyperparasitoid (Riley 1888), polyphagous (Noyes 2019).

#### *Dirhinushimalayanus* Westwood, 1836

**Distribution.** India, Iran, Japan, Malaysia, Pakistan, Philippines, Thailand, Turkmenistan, former USSR (Noyes 2019).

**Recorded interactions with *H.cunea*.** Japan (Habu 1960 as *D.luzonensis*).

**Host stage.** Pupa (Habu 1960).

**Parasitoid type.** Hyperparasitoid of tachinid, polyphagous (Habu 1960).

##### ﻿﻿Encyrtidae 跳小蜂科

#### *Exoristobiaklinoclavata* Xu, 2000

**Distribution.** China (Noyes 2019).

**Recorded interactions with *H.cunea*.** China (Yang et al. 2008).

**Host stage.** Larva or pupa (Yang et al. 2008).

**Parasitoid type.** Hyperparasitoid, endoparasitoid, gregarious (Yang et al. 2008).

**Notes.** 47 wasps were reared on average from 1 host puparium, parasitoid female to male ratio 6.2, parasitism rate 1.6% (Yang et al. 2008).

##### ﻿﻿Eulophidae 姬小蜂科

#### *Aprostocetusesurus* Riley, 1879

**Distribution.** Canada, USA (Noyes 2019).

**Recorded interactions with *H.cunea*.** USA [Washington DC (Marlatt 1903; Peck 1963 as *Syntomosphyrumesurus*).

**Host stage.** Pupa (Marlatt 1903), larva (Warren and Tadić 1967).

**Parasitoid type.** Hyperparasitoid (Marlatt 1903), polyphagous (Peck 1963).

#### *Aprostocetusmagniventer* Yang, 2003

**Distribution.** China (Noyes 2019).

**Recorded interactions with *H.cunea*.** China [Yantai City, Shandong Province] (Yang et al. 2003b).

**Host stage.** Pupa (Yang et al. 2003b).

**Parasitoid type.** Endoparasitoid, gregarious parasitism (Yang et al. 2003b).

**Notes.** Parasitoid female to male ratio 2.6, highest parasitism rate 3.5% (Yang et al. 2003b).

#### *Baryscapuscoerulescens* Ashmead, 1898

**Distribution.** Canada, USA (Noyes 2019).

**Recorded interactions with *H.cunea*.** USA (Swain 1937 as *Tctrasticlmsdotcni*), North America (Peck 1963 as *Tetrastinhuscoerulescens*).

**Host stage.** Pupa (Swain 1937).

**Parasitoid type.** Hyperparasitoid (Swain 1937; Noyes 2019).

#### *Chouioiacunea* Yang, 1989

**Distribution.** China, Iran, Italy, Japan, Korea (Noyes 2019).

**Recorded interactions with *H.cunea*.** China [Wugong County, Shaanxi Province (types); Beijing City (Yang 1989)], Italy [Cremona, Mantova, Pumenengo Bergamo (Boriani 1991), Pontirolo Nuovo, Bergamo; Eraclea, Venezia (Boriani 1994b), Iran [Guilan province (Karami et al. 2023)], South Korea (Kim et al. 2011), Turkey [Samsun region (Sullivan et al. 2011); Düzce (Avci et al. 2022)].

**Host stage.** Pupa (Yang 1989).

**Parasitoid type.** Endoparasitoid, gregarious parasitism, polyphagous, hyperparasitoid of tachinids (Yang 1989).

**Notes.** Parasitoid female to male ratio 68, the highest parasitism rate 83.2% (in lab) (Yang 1989). Average parasitism 1.9%, average clutch size 117, average parasitoid female to male ratio 44.5 (Sullivan et al. 2011).

#### *Elachertuscacoeciae* Howard, 1885

**Distribution.** Canada, USA (Noyes 2019).

**Recorded interactions with *H.cunea*.** USA [Washington DC (Howard 1885), North America (Peck 1963).

**Host stage.** Larva (Warren and Tadić 1967).

**Parasitoid type.** Primary (Howard 1885).

#### *Elachertuscidariae* Ashmead, 1898

**Distribution.** Bermuda, Canada, USA; induced to former Yugoslavia (Noyes 2019).

**Recorded interactions with *H.cunea*.** USA [New England **or** New York **or** New Jersey (Schaffner and Griswold 1934 as *E.hyphantriae* and *E.marylandicus*), North America (Warren and Tadić 1967 as *E.hyphantriae*; Burks 1979 as *E.hyphantriae* and *E.marylandicus*), USA [West Virgnia (Butler 1993)].

**Host stage.** Larva (Warren and Tadić 1967 as *E.hyphantriae*).

**Parasitoid type.** Polyphagous, ectoparasitoid (Noyes 2019).

**Notes.** Parasitism rate 0.2% (Nordin et al. 1972 as *E.hyphantriae*).

#### *Elasmusatratus* Howard, 1897

**Distribution.** Canada, USA (Noyes 2019).

**Recorded interactions with *H.cunea*.** Canada [New Brunswick; Nova Scotia] (Morris 1976a).

**Host stage.** Cocoon (Morris 1976a).

**Parasitoid type.** Hyperparasitoid (Morris 1976a), polyphagous (Noyes 2019).

**Notes.** Most prevalent hyperparasitoid (Morris 1976a).

#### *Elasmusnigrescens* Ashmead, 1895

Unavailable name, determinations made by Mr. W. H. Ashmead (Webster 1895).

**Distribution.** USA (Webster 1895).

**Recorded interactions with *H.cunea*.** USA [Warren County, Southern Ohio (Webster 1895)].

**Host stage.** Cocoon (Webster 1895).

**Parasitoid type.** Hyperparasitoid (Webster 1895).

#### *Elasmuspullatus* Howard, 1885

**Distribution.** USA (Noyes 2019).

**Recorded interactions with *H.cunea*.** USA [Ohio, Missouri, Kans (Peck 1963)].

#### *Elasmusvarius* Howard, 1885

**Distribution.** USA (Noyes 2019).

**Recorded interactions with *H.cunea*.** USA (Peck 1963).

#### *Neochrysocharishyphantriae* Yoshimoto, 1978

**Distribution.** Mexico, USA (Noyes 2019).

**Recorded interactions with *H.cunea*.** Mexico (Yoshimoto 1978).

**Host stage.** Pupa (Yoshimoto 1978).

**Parasitoid type.** Hyperparasitoid (Yoshimoto 1978).

#### *Pediobiusbruchicida* Rondani, 1872

**Distribution.** Afrotropical, Australia, Europe, Palaearctic, West Asia (Noyes 2019).

**Recorded interactions with *H.cunea*.** Europe (Noyes 2019).

**Host stage.** Pupa (Noyes 2019).

**Parasitoid type.** Facultative hyperparasitoid, endoparasitoid, gregarious (Noyes 2019).

#### *Pediobiuselasmi* (Ashmead, 1904)

**Distribution.** Australia, India, Indonesia, Malaysia, Papua New Guinea, China, Philippines, Sri Lanka (Noyes 2019).

**Recorded interactions with *H.cunea*.** China (Yang et al. 2008).

**Host stage.** Pupa (Yang et al. 2008).

**Parasitoid type.** Gregarious parasitism (Yang et al. 2008).

**Notes.** Parasitoid female to male ratio 2.8–13.6 (Yang et al. 2008); parasitism rate 2.6% (Yang et al. 2008).

#### *Pediobiuspupariae* Yang, 2015

**Distribution.** China (Noyes 2019).

**Recorded interactions with *H.cunea*.** China (Yang et al. 2015b).

**Host stage.** Pupa (Yang et al. 2015b).

**Parasitoid type.** Gregarious parasitism (Yang et al. 2015b).

#### *Pediobiuspyrgo* Walker, 1839

**Distribution.** Europe, Nearctic, Oriental, Palaearctic (Noyes 2019).

**Recorded interactions with *H.cunea*.** Europe (Noyes 2019).

**Host stage.** Pupa (Noyes 2019).

**Parasitoid type.** Primary, hyperparasitoid (Noyes 2019).

#### *Rhicnopeltecrassicornis* Nees, 1834

**Distribution.** Palaearctic, Europe, West Asia (Noyes 2019).

**Recorded interactions with *H.cunea*.** Iran [Gilan, Rezvanshahr (Yefremova et al. 2007)].

**Host stage.** Larva (Yefremova et al. 2007)

**Parasitoid type.** Gregarious parasitism, ectoparasitoid (Yefremova et al. 2007)

#### *Tetrastichomyiaclisiocampae* (Ashmead, 1894)

**Distribution.** Italy, USA (Noyes 2019).

**Recorded interactions with *H.cunea*.** Italy [Pontirolo Nuovo, Bergamo (Boriani 1994b)] (Boriani 1991 as *Tetrastichusgoidanichi*).

**Host stage.** Pupa (Boriani 1994b).

**Parasitoid type.** Endoparasitoid, hyperparasitoid (Boriani 1994b).

#### *Tetrastichuslitoreus* Yang, Qiao & Han, 2003

**Distribution.** China (Noyes 2019).

**Recorded interactions with *H.cunea*.** China [Qinhuangdao City, Hebei Province (Yang et al. 2003a)].

**Host stage.** Pupa (Yang et al. 2003a).

**Parasitoid type.** Endoparasitoid, gregarious parasitism (Yang et al. 2003a).

**Notes.** Parasitoid female to male ratio 5 (Yang et al. 2003a) and 3 (Yang et al. 2008); parasitism rate 0.1% (Yang et al. 2008).

#### *Tetrastichusnigricoxae* Yang, 2003

**Distribution.** China (Noyes 2019).

**Recorded interactions with *H.cunea*.** China [Yangling City, Shaanxi Province; Xuzhou City, Jiangsu Province (Yang and Wei 2003)].

**Host stage.** Pupa (Yang and Wei 2003).

**Parasitoid type.** Endoparasitoid, gregarious, oligophagous parasitism (Yang and Wei 2003).

**Notes.** Parasitoid female to male ratio 1.9, parasitism rate 6.2–13.4% (Yang and Wei 2003).

#### *Tetrastichusseptentrionalis* Yang, 2001

**Distribution.** China, South Korea (Noyes 2019).

**Recorded interactions with *H.cunea*.** China [Tianjin City; Dalian City, Liaoning Province; Qinhuangdao City, Hebei Province; Yantai City, Shandong Province], South Korea [Seoul] (Yang et al. 2001; Kim et al. 2011).

**Host stage.** Pupa (Yang et al. 2001).

**Parasitoid type.** Endoparasitoid, gregarious, oligophagous (Yang et al. 2001).

**Notes.** Parasitoid female to male ratio 10, the highest parasitism rate 24% (Yang et al. 2001), 3.2% (Yang et al. 2008).

#### *Tetrastichusshandongensis* Yang, 2003

**Distribution.** China (Noyes 2019).

**Recorded interactions with *H.cunea*.** China [Yantai City, Shandong Province (Yang and Wei 2003)].

**Host stage.** Pupa (Yang and Wei 2003).

**Parasitoid type.** Endoparasitoid, gregarious (Yang and Wei 2003).

**Notes.** Parasitoid female to male ratio 3.2, the parasitism rate 6.2% (Yang and Wei 2003), 3.6% (Yang et al. 2008).

#### *Trichospilusalbiflagellatus* Yang & Wang, 2015

**Distribution.** China (Noyes 2019).

**Recorded interactions with *H.cunea*.** China [Yantai City, Shandong Province (Yang et al. 2015a)].

**Host stage.** Pupa (Yang et al. 2015a).

**Parasitoid type.** Endoparasitoid, gregarious (Yang et al. 2015a).

**Notes.** Average parasitoid female to male ratio 58.56, highest parasitism rate 28.6% (Yang et al. 2001).

##### ﻿﻿Eupelmidae 旋小蜂科

#### *Eupelmusfulvipes* Förster, 1860

**Distribution.** Austria, Azerbaijan, China, France, Georgia, Germany, Hungary, Iran, Italy, Montenegro, Poland, Romania, Russia, Serbia, Spain, Turkey (Gibson and Fusu 2016; Noyes 2019; Yang et al. 2008).

**Recorded interactions with *H.cunea*.** China [Qinhuangdao City, Heibei Province (Yang et al. 2008)].

**Host stage.** Pupa (Yang et al. 2008).

**Parasitoid type.** Gregarious, endoparasitoid (Yang et al. 2008).

**Notes.** Parasitism rates 0.1%, 3 females were reared from a single pupa (Yang et al. 2008).

##### ﻿﻿Eurytomidae 广肩小蜂科

#### *Eurytomaappendigaster* Swederus, 1795

**Distribution.** Europe, North Africa, North America, China (Noyes 2019).

**Recorded interactions with *H.cunea*.** Europe (Noyes 2019).

**Host stage.** Pupa (Noyes 2019).

**Parasitoid type.** Primary, polyphagous (Noyes 2019).

#### *Eurytomagoidanichi* Boucek, 1970

**Distribution.** Europe, Iran (Noyes 2019), China (Yang et al. 2008).

**Recorded interactions with *H.cunea*.** China (Yang et al. 2008).

**Host stage.** Pupa (Yang et al. 2008)

**Parasitoid type.** Gregarious, hyperparasitoid (Yang et al. 2008).

**Notes.** Parasitoid female to male ratio 2.2, parasitism rate 22% (Yang et al. 2008).

#### *Eurytomarosae* Nees, 1834

**Distribution.** Europe, Asia, North Africa, Argentina (Noyes 2019).

**Recorded interactions with *H.cunea*.** Romania [Piatra Craiului National Park (Popescu 2006)].

**Host stage.** Pupa (Noyes 2019).

**Parasitoid type.** Primary, polyphagous (Noyes 2019).

#### *Eurytomaverticillata* (Fabricius, 1798)

**Distribution.** Europe, East Asia, North America (Noyes 2019).

**Recorded interactions with *H.cunea*.** Italy [Eraclea, Venezia (Boriani 1994b)].

**Host stage.** Pupa (Boriani 1994b).

**Parasitoid type.** Endoparasitoid, hyperparasitoid, polyphagous (Boriani 1994b).

##### ﻿﻿Perilampidae 巨胸小蜂科

#### *Perilampushyalinus* Say, 1929

**Distribution.** Canada, Cuba, Mexico, Peru, Puerto Rico, USA (Noyes 2019).

**Recorded interactions with *H.cunea*.** Canada, USA (Peck 1963).

**Host stage.** Pupa or cocoon (Smith 1912).

**Parasitoid type.** Hyperparasitoid (Smith 1912; Tripp 1962).

##### ﻿﻿Pteromalidae 金小蜂科

#### *Catolaccusaeneoviridis* Girault, 1911

**Distribution.** Bermuda, Canada, Mexico, USA, Nearctic (Noyes 2019).

**Recorded interactions with *H.cunea*.** USA [Colorado (Swain 1937)].

**Host stage.** Cocoon (Swain 1937).

**Parasitoid type.** Hyperparasitoid (Swain 1937).

#### *Coelopisthiaextenta* (Walker, 1835)

**Distribution.** Europe, Kazakhstan, Uzbekistan, USA (Noyes 2019).

**Recorded interactions with *H.cunea*.** Italy [Pontirolo Nuovo, Bergamo; Eraclea, Venezia (Boriani 1994b)].

**Host stage.** Pupa (Boriani 1994b).

**Parasitoid type.** Endoparasitoid, gregarious, hyperparasitoid (Boriani 1994b).

#### *Conomoriumamplum* (Walker, 1835)

**Distribution.** Belgium, Canary Islands, Czech, France, Germany, Greece, Hungary, Italy, Kazakhstan, Madeira, Netherlands, China, Romania, Spain, Sweden, Switzerland, Tselinograd Obl., UK, Uzbekistan (Noyes 2019).

**Recorded interactions with *H.cunea*.** Italy [Pianengo, Cremona; Bisnate, Milano, Pontirolo Nuovo, Bergamo (Boriani 1991 as *C.patulum*; Boriani 1994a)], Turkey [Samsun region (Sullivan et al. 2011)].

**Host stage.** Pupa (Boriani 1994a).

**Parasitoid type.** Endoparasitoid, gregarious (Boriani 1994a).

**Notes.** Average parasitism 0.047%, average clutch size 1.5, average parasitoid female to male ratio 0.5 (Sullivan et al. 2011).

#### *Conomoriumcuneae* Yang & Baur, 2004

**Distribution.** China (Noyes 2019).

**Recorded interactions with *H.cunea*.** China [Tianjin City; Yantai City, Shandong Province; Wugong City, Shaanxi Province, Dalian City, Liaoning Province; Qinhuangdao City, Hebei Province (Yang and Baur 2004)].

**Host stage.** Pupa (Yang and Baur 2004).

**Parasitoid type.** Endoparasitoid, gregarious (Yang and Baur 2004).

**Notes.** Parasitoid female to male ratio 7.5, highest parasitism rate 3.6–12.2% (Yang and Baur 2004), 1.2% (Yang et al. 2008).

#### *Dibrachysmaculipennis* Szelenyi, 1957

**Distribution.** Canada, Hungary, Kirgizia, Slovakia, Sweden (Noyes 2019).

**Recorded interactions with *H.cunea*.** Europe (Noyes 2019).

**Host stage.** Cocoon (Noyes 2019).

**Parasitoid type.** Hyperparasitoid (Noyes 2019).

#### *Dibrachysmicrogastri* (Bouche, 1834)

**Distribution.** Europe, Asia, North America (Noyes 2019).

**Recorded interactions with *H.cunea*.** Italy [Pontirolo Nuovo, Bergamo (Boriani 1994b as *D.boarmiae*)], China (Yang et al. 2008 as *D.cavus*), Turkey [Samsun region (Sullivan et al. 2011 as *D.boarmiae*)], North America (Warren and Tadić 1967).

**Host stage.** Pupa (Boriani 1994b), larva-pupa (Yang et al. 2008).

**Parasitoid type.** Endoparasitoid, gregarious, hyperparasitoid, polyphagous (Boriani 1994b), gregarious, hyperparasitoid (Yang et al. 2008).

**Notes.** Parasitoid female to male ratio 8.0, highest parasitism rate 0.15% (Yang et al. 2008). Average parasitism 1.2%, average clutch size 10, average female to male ratio 24 (Sullivan et al. 2011).

#### *Dirhinusanthracia* Walker, 1846

**Distribution.** Australia, India, Philippines, South Africa, China, Vietnam, Zambia (Noyes 2019).

**Recorded interactions with *H.cunea*.** South Korea (Kim et al. 2011).

**Host stage.** Pupa (Kim et al. 2011).

**Parasitoid type.** polyphagous (Noyes 2019).

#### *Hypopteromalusinimicus* Muesebeck, 1927

**Distribution.** Canada, USA (Noyes 2019).

**Recorded interactions with *H.cunea*.** Canada [New Brunswick, Nova Scotia (Morris 1976a)].

**Host stage.** Cocoon (Morris 1976a).

**Parasitoid type.** Hyperparasitoid (Morris 1976a).

#### *Hypopteromaluspercussor* Girault, 1917

**Distribution.** Canada, USA (Noyes 2019).

**Recorded interactions with *H.cunea*.** USA Colorado (Swain 1937)].

**Host stage.** Cocoon (Swain 1937).

**Parasitoid type.** Hyperparasitoid (Swain 1937).

#### *Hypopteromalustabacum* Fitch, 1864

**Distribution.** Canada, USA (Noyes 2019).

**Recorded interactions with *H.cunea*.** USA (Peck 1951).

#### *Psychophagusomnivorus* (Walker, 1835)

**Distribution.** Europe, West Asia, North Africa, North America (Noyes 2019).

**Recorded interactions with *H.cunea*.** Turkey [Samsun region (Sullivan et al. 2011), Italy (Boriani 1991), Iran [Guilan province (Karami et al. 2023)]

**Host stage.** Pupa (Sullivan et al. 2011).

**Parasitoid type.** Gregarious, polyphagous (Noyes 2019).

**Notes.** Average parasitism 6.7%, average clutch size 60, average parasitoid female to male ratio 0.92 (Sullivan et al. 2011).

#### *Pteromalusapum* Retzius, 1783

**Distribution.** Argentina, Canada, Russia, USA, Europe (Noyes 2019).

**Recorded interactions with *H.cunea*.** Europe (Warren and Tadić 1967 as *Pteromalusplaniscuta*).

**Host stage.** Pupa (Warren and Tadić 1967).

#### *Pteromalusbifoveolatus* Foerster, 1861

**Distribution.** Europe (Noyes 2019), China (Yang et al. 2008).

**Recorded interactions with *H.cunea*.** China (Yang et al. 2008).

**Host stage.** Pupa (Yang et al. 2008).

**Parasitoid type.** Gregarious parasitism (Yang et al. 2008)., polyphagous (Noyes 2019).

**Notes.** Parasitoid female to male ratio 5.5, highest parasitism rate 0.2% (Yang et al. 2008).

#### *Pteromalusegregious* Foerster, 1841

**Distribution.** Canada, Germany, Hungary, USA (Noyes 2019).

**Recorded interactions with *H.cunea*.** Europe (Noyes 2019).

#### *Pteromalusphycidis* Ashmead, 1898

**Distribution.** Canada, USA (Noyes 2019).

**Recorded interactions with *H.cunea*.** North America (Noyes 2019).

**Parasitoid type.** Hyperparasitoid (Noyes 2019).

#### *Trichomalopsiscotesiae* Yang, 2015

**Distribution.** China (Noyes 2019).

**Recorded interactions with *H.cunea*.** China (Yang et al. 2015b).

**Host stage.** Larva (Yang et al. 2015b).

**Parasitoid type.** Hyperparasitoid (Yang et al. 2015b).

#### *Trichomalopsisgenalis* (Graham, 1969)

**Distribution.** Europe (Noyes 2019), China (Yang et al. 2008).

**Recorded interactions with *H.cunea*.** China (Yang et al. 2008).

**Host stage.** 4^th^ instar larva (Yang et al. 2008).

**Parasitoid type.** Gregarious, polyphagous (Yang et al. 2008).

**Notes.** Parasitoid female to male ratio 1.6, parasitism rate 0.2% (Yang et al. 2008).

#### *Trichomalopsisgermanica* (Graham, 1969)

**Distribution.** Germany, Sweden (Noyes 2019), China (Yang et al. 2008).

**Recorded interactions with *H.cunea*.** China (Yang et al. 2008 as *T.germanicus*).

**Host stage.** Pupa (Yang et al. 2008)

**Parasitoid type.** Gregarious, hyperparasitoid (Yang et al. 2008).

**Notes.** Parasitoid female to male ratio 2, highest parasitism rate 15% (Yang et al. 2008).

#### *Trichomalopsishemiptera* Walker, 1835

**Distribution.** Europe, North America, East Asia (Noyes 2019).

**Recorded interactions with *H.cunea*.** USA [New England (Peck 1963 as *Eupterotnalushernipterus*, laboratory rearing)].

**Parasitoid type.** Hyperparasitoid (Noyes 2019).

##### ﻿﻿Torymidae 长尾小蜂科

#### *Monodontomerusaeneus* Fonscolombe, 1832

**Distribution.** Europe, Iran, Kazakhstan, China, USA, Chile (Noyes 2019).

**Recorded interactions with *H.cunea*.** Europe (Noyes 2019).

**Parasitoid type.** Polyphagous (Noyes 2019).

#### *Monodontomerusaereus* Walker, 1834

**Distribution.** Europe, Asia, North Africa, North America (Noyes 2019).

**Recorded interactions with *H.cunea*.** North America, Europe (Warren and Tadić 1967).

**Host stage.** Larva (Warren and Tadić 1967).

**Parasitoid type.** Polyphagous (Noyes 2019).

#### *Monodontomerusdentipes* Dalman, 1820

**Distribution.** Europe, Asia, North America (Noyes 2019).

**Recorded interactions with *H.cunea*.** Europe (Noyes 2019).

**Parasitoid type.** Polyphagous (Noyes 2019).

#### *Monodontomerusminor* (Ratzeburg, 1848)

**Distribution.** Europe, Asia, North America (Noyes 2019).

**Recorded interactions with *H.cunea*.** Italy [Eraclea, Venezia (Boriani 1994b)], China (Yang et al. 2008).

**Host stage.** Pupa (Boriani 1994b), larva-pupa (Yang et al. 2008).

**Parasitoid type.** Endoparasitoid, gregarious, hyperparasitoid, polyphagous (Boriani 1994b), hyperparasitoid (Yang et al. 2008).

**Notes.** Parasitoid female to male ratio 1.4, highest parasitism rate 0.15% (Yang et al. 2008).

##### ﻿﻿Trichogrammatidae 赤眼蜂科

#### *Trichogrammabrassicae* Bezdenko, 1968

**Distribution.** Europe, Asia, Australia, North America (Noyes 2019).

**Recorded interactions with *H.cunea*.** Turkey [Düzce (Avci et al. 2022)].

**Host stage.** Egg (Avci et al. 2022).

**Parasitoid type.** Polyphagous (Noyes 2019).

#### *Trichogrammacacaeciae* Marchal, 1927

**Distribution.** Europe, Asia, North America, South America (Noyes 2019).

**Recorded interactions with *H.cunea*.** Moldova (Plugaru 1979).

**Host stage.** Egg (Plugaru 1979).

**Parasitoid type.** Polyphagous (Noyes 2019).

#### *Trichogrammadendrolimi* Matsumura, 1926

**Distribution.** Europe, Asia, Chile (Noyes 2019).

**Recorded interactions with *H.cunea*.** Asia (Warren and Tadić 1967), South Korea (Noyes 2019), China [Beijing, tested in lab, unpublished data, CLM].

**Host stage.** Egg (Warren and Tadić 1967; Noyes 2019).

**Parasitoid type.** Polyphagous, primary (Noyes 2019).

#### *Trichogrammaevanescens* Westwood, 1833

**Distribution.** Europe, Asia, North America, South America (Noyes 2019).

**Recorded interactions with *H.cunea*.** Europe (Warren and Tadić 1967).

**Host stage.** Egg (Warren and Tadić 1967).

**Parasitoid type.** Polyphagous (Noyes 2019).

#### *Trichogrammaminutum* Riley, 1871

**Distribution.** Europe, Asia, Africa, North America, South America (Noyes 2019).

**Recorded interactions with *H.cunea*.** Europe (Warren and Tadić 1967), Italy (Viggiani and Laudonia 1989).

**Host stage.** Egg (Warren and Tadić 1967).

**Parasitoid type.** Polyphagous (Noyes 2019).

#### *Trichogrammaostriniae* Pang & Chen, 1974

**Distribution.** China, Japan, South Korea, South Africa, USA (Noyes 2019).

**Recorded interactions with *H.cunea*.** China [Beijing, tested in lab, unpublished data, CLM].

**Host stage.** Egg (unpublished data).

**Parasitoid type.** Polyphagous (Noyes 2019).

#### *Trichogrammapiceum* Dyurich, 1987

**Distribution.** Italy, Moldova (Noyes 2019).

**Recorded interactions with *H.cunea*.** Italy (Noyes 2019).

**Host stage.** Egg (Noyes 2019).

**Parasitoid type.** Polyphagous (Noyes 2019).

##### ﻿﻿Scelionidae 缘腹细蜂科

#### *Telenomuschloropus* (Thomson, 1861)

**Distribution.** Ukraine, Turkey, UK, Russia, Moldavia, Kazakhstan, Georgia, Kazakhstan, Far East, France, Hungary, Japan, Spain, Sweden, USA, Ireland, Iran (Samin et al. 2010).

**Recorded interactions with *H.cunea*.** Europe (Warren and Tadić 1970 as *Telenomusmayri*).

**Host stage.** Egg (Warren and Tadić 1970).

#### *Telenomusbifidus* Riley, 1887

**Distribution.** USA (Riley 1887a).

**Recorded interactions with *H.cunea*.** USA [Washington, D.C. (Riley 1887a)].

**Host stage.** Egg (Riley 1887a).

**Parasitoid type.** Endoparasitoid, oligophagous (Riley 1887b),

**Notes.** Parasitize eggs of first and second generations of fall webworm. Useful natural enemy (Riley 1887a).

##### ﻿﻿Diptera

﻿**Tachinidae** 寄蝇科

#### Archytas (Nemochaeta) aterrimus (Robineau-Desvoidy, 1830)

**Distribution.** Canada, USA, Mexico (O’Hara et al. 2020).

**Recorded interactions with *H.cunea*.** North America (Sullivan and Ozman-Sullivan 2012).

#### *Bactromyiaaurulenta* (Meigen, 1824)

**Distribution.** China, Europe, Japan, South Korea, Russia, Transcaucasia (O’Hara et al. 2020).

**Recorded interactions with *H.cunea*.** Japan [Honshu, Ibaraki (Tschorsnig 2017)].

#### *Bessaparallela* (Meigen, 1824)

**Distribution.** China, Europe, Japan, Mongolia, Russia, Armenia (O’Hara et al. 2020).

**Recorded interactions with *H.cunea*.** Japan [Tsukuba (Watanabe 2005)], Serbia, Hungary, Japan, Montenegro, Moldova [Kishinev], Russia [European part], Japan [Honshu, Tokyo, Fuchu, Nara] (Tschorsnig 2017). Asia, Europe (Sullivan and Ozman-Sullivan 2012 as *Bessaselecta*, *Ptychomyiaselecta*).

**Host stage.** Pupa (Warren and Tadić 1967 as *B.fugax* and *B.selecta*).

#### *Blepharipasericariae* (Rondani, 1870)

**Distribution.** Palaearctic: China, Japan (O’Hara et al. 2020).

**Recorded interactions with *H.cunea*.** Japan [Honshu, Tokyo] (Tschorsnig 2017).

#### *Blepharipazebina* (Walker, 1849)

**Distribution.** China, Japan, North Korea, South Korea, Russia, India, Myanmar, Nepal, Sri Lanka, Thailand (O’Hara et al. 2020).

**Recorded interactions with *H.cunea*.** China [Wugong Conty, Shaanxi Province (Ran and Zhao 1989)].

**Host stage.** Pupa (Ran and Zhao 1989).

**Parasitoid type.** Solitary, polyphagous (Ran and Zhao 1989).

#### *Blondeliaeufitchiae* (Townsend, 1892)

**Distribution.** Canada, USA (O’Hara et al. 2020).

**Recorded interactions with *H.cunea*.** USA [Colorado (Swain 1937 as *Masiceraeufitchiae*)], North America (Sullivan and Ozman-Sullivan 2012).

**Host stage.** Larva (Warren and Tadić 1967).

#### *Blondeliahyphantria* (Tothill, 1922)

**Distribution.** Canada, USA, China (O’Hara et al. 2020).

**Recorded interactions with *H.cunea*.** Canada (Tothill 1922 as *Lydellahyphantriae*), USA [Colorado] (Swain 1937 as *Anetiahyphantriae* and *Lydellahyphantriae*), North America (Sullivan and Ozman-Sullivan 2012).

**Host stage.** Larva-pupa (Tothill 1922), larva (Warren and Tadić 1967).

**Parasitoid type.** Solitary (Tothill 1922).

**Notes.** Maggot travels from one feeding area to another (Tothill 1922).

#### *Blondelianigripes* (Fallén, 1810)

**Distribution.** Central Asia, China, Japan, South Korea, Iran, Mongolia, Russia, Transcaucasia, Europe (O’Hara et al. 2020).

**Recorded interactions with *H.cunea*.** Europe (Sullivan and Ozman-Sullivan 2012), Hungary, Russia (Tschorsnig 2017).

#### *Blondeliaobconica* (Walker, 1853)

**Distribution.** USA (O’Hara et al. 2020).

**Recorded interactions with *H.cunea*.** North America (Sullivan and Ozman-Sullivan 2012 as *Tachinaobconica*).

#### *Cadurcia* sp.

**Distribution.** Europe (Sullivan and Ozman-Sullivan 2012).

**Recorded interactions with *H.cunea*.** Europe (Sullivan and Ozman-Sullivan 2012).

#### *Carceliabombylans* Robineau-Desvoidy, 1830

**Distribution.** China, Europe, Japan, Russia, Azerbaijan (O’Hara et al. 2020).

**Recorded interactions with *H.cunea*.** Italy (Boriani 1991). Serbia, Italy [Lombardia, Mantua] (Tschorsnig 2017).

#### *Carceliagnava* (Meigen, 1824)

**Distribution.** China, Europe, Japan, South Korea, Russia, Armenia (O’Hara et al. 2020).

**Recorded interactions with *H.cunea*.** South Korea (Tschorsnig 2017).

#### *Carceliakockiana* (Townsend, 1927)

**Distribution.** China (Ran and Zhao 1989), India, Indonesia, Malaysia, Philippines, Papua New Guinea (O’Hara et al. 2020).

**Recorded interactions with *H.cunea*.** China [Wugong County, Shaanxi Province (Ran and Zhao 1989)].

**Host stage.** Pupa (Ran and Zhao 1989).

**Parasitoid type.** Solitary, polyphagous (Ran and Zhao 1989).

#### *Carceliamatsukarehae* (Shima, 1969)

**Distribution.** China, Japan, Russia (O’Hara et al. 2020).

**Recorded interactions with *H.cunea*.** China [Shandong (Tschorsnig 2017)].

#### *Carceliaprotuberans* (Aldrich & Webber, 1924)

**Distribution.** Canada, USA (O’Hara et al. 2020).

**Recorded interactions with *H.cunea*.** USA [Colorado (Swain 1937 as *Zenilliaprotuberans*)], North America (Sullivan and Ozman-Sullivan 2012).

**Host stage.** Larva (Warren and Tadić 1967).

#### *Carceliasumatrana* Townsend, 1927

**Distribution.** China, Japan, Russia, Indonesia, Malaysia, Sri Lanka (O’Hara et al. 2020).

**Recorded interactions with *H.cunea*.** Japan [Honshu, Tokyo, Fuchu City (Tschorsnig 2017)].

#### *Ceromasiaauricaudata* Townsend, 1908

**Distribution.** Canada, USA (O’Hara et al. 2020).

**Recorded interactions with *H.cunea*.** North America (Sullivan and Ozman-Sullivan 2012).

#### *Ceromasiarubrifrons* (Macquart, 1834)

**Distribution.** Uzbekistan, China, Europe, Japan, Israel, Mongolia, Morocco, Russia, Transcaucasia (O’Hara et al. 2020).

**Recorded interactions with *H.cunea*.** Moldova (Tschorsnig 2017).

#### *Chetogenaclaripennis* (Macquart, 1848)

**Distribution.** Canada, USA, Puerto Rico, Mexico, Venezuela (O’Hara et al. 2020).

**Recorded interactions with *H.cunea*.** USA [Colorado (Swain 1937 as *Phoroceraclaripennis*)], North America (Warren and Tadić 1967 as *Euphoroceraclaripenn*, Sullivan and Ozman-Sullivan 2012).

**Host stage.** Larva (Warren and Tadić 1967).

#### *Chetogenascutellaris* (van der Wulp, 1890)

**Distribution.** USA, Venezuela (O’Hara et al. 2020).

**Recorded interactions with *H.cunea*.** USA [Colorado (Swain 1937 as *Phorocerafloridensis*)], North America (Warren and Tadić 1967 as *Euphorocerafloridensis*, Sullivan and Ozman-Sullivan 2012), Mexico [Tamaulipas (Kasparyan and Pinson 2007)].

#### *Clemelispullata* (Meigen, 1824)

**Distribution.** China, Europe, Israel, Mongolia, Morocco, Russia, Armenia (O’Hara et al. 2020).

**Recorded interactions with *H.cunea*.** Moldova [Cahul (Tschorsnig 2017)].

#### *Compsiluraconcinnata* (Meigen, 1824)

**Distribution.** Canada, USA, China, Europe, Japan, Kazakhstan, North Korea, South Korea, Iran, Israel, Lebanon, Algeria, Egypt, Morocco, Russia, Armenia, Azerbaijan, Nigeria, South Africa, India, Indonesia, Malaysia, Australia, Papua New Guinea (O’Hara et al. 2020).

**Recorded interactions with *H.cunea*.** Canada (Morris 1976b), Italy (Boriani 1991), Turkey [Samsun Province (Sullivan et al. 2012)], Iran [Guilan province (Karami et al. 2023)], Japan [Tsukuba (Watanabe 2005)], China (Yang et al. 2008). Austria [Burgenland, Weiden], Serbia [Vojvodina], Hungary, Romania [Bukarest], Ukraine [Transcarpathia], Slovakia [Gabčíkovo, Nitra env.], Japan [Honshu, Gunma], France [Gironde and/or Landes], Bulgaria [Silistra], Turkey [Samsun], Moldova, Italy [Lombardia, Ponte Merlano, Emilia-Romagna, Mantova], Russia, [Voronezh Region], Azerbaijan [Guba-Khachmaz Region], China [Shangdong, Hebei, Liaoning] (Tschorsnig 2017).

**Host stage.** Pupa and larva (Warren and Tadić 1967), pupa (Sullivan et al. 2012).

**Parasitoid type.** Endoparasitoid, solitary or gregarious, polyphagous (Yang et al. 2008).

**Notes.** Parasitism rate is higher for wandering *H.cunea* larvae than in feeding larvae (Watanabe 2005). Parasitoid female to male ratio 1.5 (Yang et al. 2008); parasitism rate 2% (Yang et al. 2008). Female injects fully incubated eggs directly into the host haemocoel. Parasitism 0.14% in Samsun (Sullivan et al. 2012).

#### *Drinofacialis* (Townsend, 1928)

**Distribution.** China, India, Indonesia, Malaysia, Philippines, Sri Lanka, Thailand (O’Hara et al. 2020).

**Recorded interactions with *H.cunea*.** China [Wugong Conty, Shaanxi Province (Ran and Zhao 1989)].

**Host stage.** Pupa (Ran and Zhao 1989).

**Parasitoid type.** Solitary, polyphagous (Ran and Zhao 1989).

#### *Drinoinconspicua* (Meigen, 1830)

**Distribution.** China, Europe, Algeria, Egypt, Russia, Armenia, Azerbaijan, Georgia (O’Hara et al. 2020).

**Recorded interactions with *H.cunea*.** Europe (Sullivan and Ozman-Sullivan 2012 as *Drina incospicua*, *Sturmiainconspicua*), Serbia [Vojvodina], Hungary (Tschorsnig 2017).

**Host stage.** Larva (Warren and Tadić 1967).

#### *Drinoinconspicuoides* (Baranov, 1932)

**Distribution.** China, Japan (O’Hara et al. 2020).

**Recorded interactions with *H.cunea*.** Japan [Tsukuba (Watanabe 2005)].

**Notes.** Parasitism rate is higher in wandering *H.cunea* larvae than in feeding larvae (Watanabe 2005).

#### *Eurysthaeascutellaris* (Robineau-Desvoidy, 1849)

**Distribution.** China, Europe, Japan, Russia, Armenia, Azerbaijan (O’Hara et al. 2020).

**Recorded interactions with *H.cunea*.** Serbia (Tschorsnig 2017).

#### *Exoristafasciata* (Fallén, 1820)

**Distribution.** China, Europe, Mongolia, Egypt, Russia, Transcaucasia (O’Hara et al. 2020).

**Recorded interactions with *H.cunea*.** China [Wugong Conty, Shaanxi Province (Ran and Zhao 1989); Dandong City, Liaoning Province (Shu and Yu 1985)].

**Host stage.** Pupa and larva (Warren and Tadić 1967), Pupa (Ran and Zhao 1989).

**Parasitoid type.** Solitary, polyphagous (Ran and Zhao 1989).

#### *Exoristajaponica* (Townsend, 1909)

**Distribution.** China, Japan, North Korea, South Korea, India, Nepal, Vietnam (O’Hara et al. 2020).

**Recorded interactions with *H.cunea*.** China [Dandong City, Liaoning Province (Shi 1981); Wugong Conty, Shaanxi Province (Ran and Zhao 1989)], China (Yang et al. 2008), Japan [Tsukuba (Watanabe 2005)]. Japan [Honshu, Saitama, Tokyo, Kanazawa], South Korea (Tschorsnig 2017).

**Host stage.** Larva to pupa (Ran and Zhao 1989).

**Parasitoid type.** Solitary, polyphagous (Ran and Zhao 1989; Yang et al. 2008).

**Notes.** Female oviposits on surface of host. Parasitism rate is higher for wandering *H.cunea* larvae than in feeding larvae (Watanabe 2005); parasitoid female to male ratio 1.5 (Yang et al. 2008); parasitism rate 10% (Shu and Yu 1985), 4–15.7% (Yang et al. 2008).

#### *Exoristalarvarum* (Linnaeus, 1758)

**Distribution.** Canada, USA, Tajikistan, Turkmenistan, China, Europe, Japan, North Korea, Iran, Israel, Saudi Arabia, Mongolia, Egypt, Russia, India (O’Hara et al. 2020).

**Recorded interactions with *H.cunea*.** Italy (Boriani 1991), Iran [Guilan province (Karami et al. 2023)], Turkey [Düzce (Avci et al. 2022)]. Austria [Burgenland, Weiden], Serbia [Vojvodina], Hungary, Romania, Turkey, Moldova, Italy [Lombardia, Ponte Merlano], Russia, Azerbaijan (Tschorsnig 2017).

**Host stage.** Pupa (Warren and Tadić 1967).

#### *Exoristarustica* (Fallén, 1810)

**Distribution.** China, Europe, Kazakhstan, North Korea, South Korea, Israel, Mongolia, Egypt, Russia, Transcaucasia, Thailand (O’Hara et al. 2020).

**Recorded interactions with *H.cunea*.** Hungary (Tschorsnig 2017).

#### *Exoristasegregata* (Rondani, 1859)

**Distribution.** Turkmenistan, Uzbekistan, Europe, Iran, Israel, Lebanon, Mongolia, Algeria, Canary Islands, Egypt, Morocco, Tunisia, Russia, Armenia, Azerbaijan, Georgia (O’Hara et al. 2020).

**Recorded interactions with *H.cunea*.** Serbia, Hungary (Tschorsnig 2017).

#### *Exoristasorbillans* (Wiedemann, 1830)

**Distribution.** Tajikistan, China, Hungary, Romania, Ukraine, Bulgaria, Greece, Italy, Serbia, Spain, Turkey, Austria, France, Japan, South Korea, Iran, Israel, Mongolia, Canary Islands, Egypt, Russia, Cameroon, D.R. Congo, Kenya, Malawi, Nigeria, Sierra Leone, Uganda, India, Australia, Lord Howe Island, Papua New Guinea (O’Hara et al. 2020).

**Recorded interactions with *H.cunea*.** China [Shandong (Tschorsnig 2017)].

#### *Exoristaxanthaspis* (Wiedemann, 1830)

**Distribution.** Tajikistan, Turkmenistan, Uzbekistan, China, Europe, Kazakhstan, South Korea, Israel, Saudi Arabia, Mongolia, Egypt, Russia, Transcaucasia, Africa, U.A. Emirates, Yemen, India, Indonesia, Japan, Thailand (O’Hara et al. 2020).

**Recorded interactions with *H.cunea*.** Serbia [Vojvodina], Serbia, Hungary, Romania, Bulgaria [Silistra region], Russia (Tschorsnig 2017).

**Host stage.** Pupa and larva (Warren and Tadić 1967 as *E.fallax*).

#### *Goniabimaculata* Wiedemann, 1819

**Distribution.** Tajikistan, Turkmenistan, Uzbekistan, China, Europe, Iran, Israel, “Palestine”, Saudi Arabia, Algeria, Canary Islands, Egypt, Tunisia, Azerbaijan (O’Hara et al. 2020).

**Recorded interactions with *H.cunea*.** Serbia [Vojvodina (Tschorsnig 2017)].

**Host stage.** Larva (Warren and Tadić 1967).

#### *Hyphantrophagablanda* (Osten Sacken, 1887)

**Distribution.** Canada, USA (O’Hara et al. 2020).

**Recorded interactions with *H.cunea*.** Canada [Quebec (Beaulne 1939 as *Zenillablanda*)], Canada (Morris 1976b as *Eusisyropablanda*).

**Host stage.** Larva (Warren and Tadić 1967), larva of later instar (Morris 1976b).

**Parasitoid type.** Solitary, endoparasitoid, koinobiont (Morris 1976b).

#### *Hyphantrophagahyphantriae* (Townsend, 1891)

**Distribution.** Canada, USA (O’Hara et al. 2020).

**Recorded interactions with *H.cunea*.** USA [Colorado (Swain 1937 as *Hyphantrophagadesmiae*)], North America (Warren and Tadić 1967 as *Hyphantrophagadesmiae*, Sullivan and Ozman-Sullivan 2012).

**Host stage.** Larva (Warren and Tadić 1967).

#### *Hyphantrophagavirilis* (Aldrich & Webber, 1924)

**Distribution.** Canada, USA, Costa Rica, Mexico (O’Hara et al. 2020).

**Recorded interactions with *H.cunea*.** USA [Colorado (Swain 1937 as *Zenilliavirilis*)], North America (Warren and Tadić 1967 as *Eusisyropavirilis*, Sullivan and Ozman-Sullivan 2012).

**Host stage.** Larva (Warren and Tadić 1967).

#### *Hystriciaabrupta* (Wiedemann, 1830)

**Distribution.** Canada, USA, Mexico (O’Hara et al. 2020).

**Recorded interactions with *H.cunea*.** USA [Colorado (Swain 1937 as *Bombyliopsisabrupta*)], North America (Warren and Tadić 1967 as *Bombyliopsisabrupta*, Sullivan and Ozman-Sullivan 2012).

**Host stage.** Larva (Warren and Tadić 1967).

#### *Isosturmiapicta* (Baranov, 1932)

**Distribution.** China, Japan, South Korea, India, Malaysia, Nepal, Sri Lanka (O’Hara et al. 2020).

**Recorded interactions with *H.cunea*.** South Korea (Tschorsnig 2017).

#### *Kuwanimyiaconspersa* Townsend, 1916

**Distribution.** China, Japan (O’Hara et al. 2020).

**Recorded interactions with *H.cunea*.** Japan [Honshu, Tokyo, Asukayama (Tschorsnig 2017)].

#### *Lespesiaaletiae* (Riley, 1879)

**Distribution.** Canada, USA, Puerto Rico, Costa Rica, Honduras, Mexico, Argentina, Brazil,

Uruguay (O’Hara et al. 2020).

**Recorded interactions with *H.cunea*.** USA [Colorado (Swain 1937 as *Achaetoneuraaletia*)], North America (Warren and Tadić 1967; Sullivan and Ozman-Sullivan 2012).

**Host stage.** Larva (Swain 1937; Warren and Tadić 1967).

**Parasitoid type.** Solitary, endoparasitoid.

#### *Lespesiaarchippivora* (Riley, 1871)

**Distribution.** Canada, USA, Cuba, Puerto Rico, Guadeloupe, Trinidad and Tobago, Guatemala, Honduras, Mexico, Nicaragua, Panama, Argentina, Brazil, Colombia, Peru, Uruguay, Venezuela (O’Hara et al. 2020).

**Recorded interactions with *H.cunea*.** North America (Sullivan and Ozman-Sullivan 2012).

#### *Lespesiafrenchii* (Williston, 1889)

**Distribution.** Canada, USA (O’Hara et al. 2020).

**Recorded interactions with *H.cunea*.** USA [Colorado (Swain 1937 as *Achaetoneurafrenchii*)].

**Host stage.** Larva (Swain 1937; Warren and Tadić 1967).

**Parasitoid type.** Solitary, endoparasitoid.

#### *Myiopharusfloridensis* (Townsend, 1892)

**Distribution.** USA, Jamaica, Puerto Rico, Mexico (O’Hara et al. 2020).

**Recorded interactions with *H.cunea*.** North America (Warren and Tadić 1967 as *Euphorocerofloridensis*).

**Host stage.** Larva (Warren and Tadić 1967).

#### *Nemoraeapellucida* (Meigen, 1824)

**Distribution.** China, Europe, Japan, Kazakhstan, South Korea, Iran, Algeria, Russia, Georgia (O’Hara et al. 2020).

**Recorded interactions with *H.cunea*.** Italy (Boriani 1991), Turkey [Samsun Province (Sullivan et al. 2012)]. France [Gironde and/or Landes], Turkey [Tekirdağ, Samsun, Saricakaya, Adapazari], Italy [Emilia-Romagna, Mantova, Lombardia, Mantua, Veneto, Pavia], Romania [Bukarest] (Tschorsnig 2017).

**Host stage.** Pupa (Sullivan et al. 2012).

**Parasitoid type.** Endoparasitoid, solitary, polyphagous.

**Notes.** Female deposits fully developed eggs near hosts, and eggs develop into planidium-type larvae that mount and enter the hosts. Parasitism rate 2.4–19.4% in Samsun (Sullivan et al. 2012).

#### *Nileahortulana* (Meigen, 1824)

**Distribution.** China, Europe, Japan, Russia (O’Hara et al. 2020).

**Recorded interactions with *H.cunea*.** Japan (Tschorsnig 2017).

#### *Palespavida* (Meigen, 1824)

**Distribution.** Turkmenistan, China, Europe, Japan, Kazakhstan, Iran, Israel, Mongolia, Morocco, Russia, Armenia, Azerbaijan, Georgia (O’Hara et al. 2020).

**Recorded interactions with *H.cunea*.** China [Dandong City, Liaoning Province (Shu and Yu 1985 as *Centophorocerapavida*); Wugong Conty, Shaanxi Province (Ran and Zhao 1989)], Japan [Tsukuba (Watanabe 2005)], Turkey [Düzce (Avci et al. 2022)]. Japan [Tokyo, Koto, Tachikawa, Honshu], Serbia [Vojvodina], Hungary, Montenegro, Moldova, China [Liaoning], Russia, Ukraine [Crimea, Yarkoe Pole], Turkey [Samsun], Italy [Pavia, Emilia-Romagna, Bologna, Lombardia] (Tschorsnig 2017).

**Host stage.** Larva (Warren and Tadić 1967), pupa (Ran and Zhao 1989).

**Parasitoid type.** Solitary, polyphagous (Ran and Zhao 1989).

#### *Panzeriaaldrichi* (Townsend, 1892)

**Distribution.** Canada, USA (O’Hara et al. 2020).

**Recorded interactions with *H.cunea*.** USA [Colorado (Swain 1937 as *Varichaetaaldrichi*)], North America (Warren and Tadić 1967 as *Mericiaaldrichi*, Sullivan and Ozman-Sullivan 2012).

**Host stage.** Larva (Warren and Tadić 1967).

#### *Panzeriaampelus* (Walker, 1849)

**Distribution.** Canada, USA (O’Hara et al. 2020).

**Recorded interactions with *H.cunea*.** USA [Colorado (Swain 1937 as *Panzeriaradicum*, *Ernestiaampelus*)], North America (Warren and Tadić 1967 as *Ernestiaampelus*, Sullivan and Ozman-Sullivan 2012), Canada (Morris 1976b as *Mericiaampelus*).

**Host stage.** Larva (Warren and Tadić 1967), Later instar larvae (Morris 1976b).

**Parasitoid type.** Solitary, endoparasitoid, koinobiont (Morris 1976b).

**Notes.** Univoltine in Canada, female deposits larvae on foliage near *H.cunea* colonies, parasitoid larvae attack host larvae they contact (Morris 1976b).

#### *Panzeriaarcuata* (Tothill, 1921)

**Distribution.** Canada, USA (O’Hara et al. 2020).

**Recorded interactions with *H.cunea*.** North America (Sullivan and Ozman-Sullivan 2012).

#### *Panzeriajohnsoni* (Tothill, 1921)

**Distribution.** Canada, USA (O’Hara et al. 2020).

**Recorded interactions with *H.cunea*.** USA [Colorado (Swain 1937 as *Ernestiajohnsoni*)], North America (Warren and Tadić 1967 as *Mericiajohnsoni*, Sullivan and Ozman-Sullivan 2012).

**Host stage.** Larva (Warren and Tadić 1967).

#### *Patelloaleucaniae* (Coquillett, 1897)

**Distribution.** Canada, USA (O’Hara et al. 2020).

**Recorded interactions with *H.cunea*.** North America (Warren and Tadić 1967; Sullivan and Ozman-Sullivan 2012).

**Host stage.** Larva (Warren and Tadić 1967).

#### *Pseudogoniaparisiaca* (Robineau-Desvoidy, 1851)

**Distribution.** China, Europe, Kazakhstan, Russia, Transcaucasia (O’Hara et al. 2020).

**Recorded interactions with *H.cunea*.** Italy (Tschorsnig 2017).

#### *Senometopiaprima* (Baranov, 1931)

**Distribution.** China, Japan, India, Indonesia (O’Hara et al. 2020).

**Recorded interactions with *H.cunea*.** Japan (Tschorsnig 2017).

#### *Sturmiabella* (Meigen, 1824)

**Distribution.** Central Asia, China, Europe, Japan, South Korea, Middle East, Morocco, Russia, Armenia, Georgia, Nepal, New Caledonia, Solomon Islands (O’Hara et al. 2020).

**Recorded interactions with *H.cunea*.** Japan (Tschorsnig 2017).

#### *Tachinapraeceps* Meigen, 1824

**Distribution.** Kyrgyzstan, Turkmenistan, Uzbekistan, China, Europe, Kazakhstan, Iran, Israel, Mongolia, North Africa, Russia, Armenia, Azerbaijan (O’Hara et al. 2020).

**Recorded interactions with *H.cunea*.** Moldova (Tschorsnig 2017).

#### *Thelairanigripes* (Fabricius, 1794)

**Distribution.** China, Europe, Japan, North Korea, South Korea, Iran, Russia (O’Hara et al. 2020).

**Recorded interactions with *H.cunea*.** Russia, Serbia (Tschorsnig 2017).

**Host stage.** Larva (Warren and Tadić 1967).

#### *Winthemia* sp.

**Distribution.** North America.

**Recorded interactions with *H.cunea*.** USA [Colorado (Swain 1937)], North America (Sullivan and Ozman-Sullivan 2012).

#### *Zenilliadolosa* (Meigen, 1824)

**Distribution.** China, Europe, Japan, South Korea, Russia (O’Hara et al. 2020).

**Recorded interactions with *H.cunea*.** Japan [Tsukuba (Watanabe 2005)].

#### *Zenillialibatrix* (Panzer, 1797)

**Distribution.** China, Europe, Japan, Iran, Russia, Armenia (O’Hara et al. 2020).

**Recorded interactions with *H.cunea*.** Italy (Boriani 1991). Italy, Japan (Tschorsnig 2017).

**Host stage.** Larva (Warren and Tadić 1967).

##### ﻿﻿Odiniidae 树创蝇科

#### ? *Odiniamaculata* (Meigen, 1830)

**Distribution.** North America.

**Recorded interactions with *H.cunea*.** Canada [Quebec (Beaulne 1939)].

**Notes.**Gaimari and Mathis (2011) cataloged *O.maculata* as a synonym of *O.trinotata* Robineau-Desvoidy, 1830, but the latter is only distributed in Europe in their catalogue; Beaulne (1939) noted *O.maculata* was a parasitoid of *H.cunea*, which may be a misidentification.

##### ﻿﻿Sarcophagidae 麻蝇科

#### *Sarcophagacarnaria* (Linnaeus, 1758)

**Distribution.** Palaearctic (Pape 1996).

**Recorded interactions with *H.cunea*.** Europe (Warren and Tadić 1967).

**Host stage.** Pupa (Warren and Tadić 1967).

**Parasitoid type.** Polyphagous.

##### ﻿﻿Muscidae 蝇科

#### *Muscadomestica* Linnaeus, 1758

**Distribution.** Originated from central Asia, but now occurs on all inhabited continents, Europe, Asia, Africa, Australasia, Arctic, Americas (Hewitt 2011).

**Recorded interactions with *H.cunea*.** Europe (Warren and Tadić 1967).

**Host stage.** Pupa and Larva (Warren and Tadić 1967).

**Parasitoid type.** Polyphagous.

#### *Muscinastabulans* (Fallén, 1817)

**Distribution.** Cosmopolitan (de Carvalho et al. 2005).

**Recorded interactions with *H.cunea*.** Europe (Warren and Tadić 1967).

**Host stage.** Larva (Warren and Tadić 1967).

**Parasitoid type.** Polyphagous.

##### ﻿﻿Phoridae 蚤蝇科

#### *Megaseliascalaris* (Loew, 1866)

**Distribution.** Europe, Africa, Asia, Americas (Karami et al. 2023).

**Recorded interactions with *H.cunea*.** Iran [Guilan province (Karami et al. 2023)].

**Host stage.** Pupa (Karami et al. 2023).

**Parasitoid type.** Polyphagous (Karami et al. 2023).

## ﻿﻿Discussion

The diversity of *H.cunea* predators and parasitoids differs among its native and invaded ranges. In North America, where *H.cunea* is native, 128 predators and 76 parasitoids have been reported. *Hyphantriacunea* is not considered a major pest in North America, which is likely attributed, in part, to its long co-evolutionary history with its natural enemies such as birds, spiders, and insects (Schowalter and Ring 2017). In the Eastern Hemisphere, 78 predators and 62 parasitoids have been reported in Asia, and 88 predators and 68 parasitoids have been reported in Europe. Currently, *H.cunea* is rarely reported as causing significant damage in some countries such as Hungary, Italy, and Japan: *H.cunea* was introduced into these countries in the 1940’s, and it is possible that native enemies have had sufficient time to adapt to *H.cunea* and help control its outbreaks. However, in newly invaded countries such as China, Iran, and Turkey, *H.cunea* outbreaks frequently occur, possibly because native natural predators in these countries are still adapting to *H.cunea*.

## ﻿Additional information

### Author ORCIDs

Liang Ming Cao https://orcid.org/0000-0002-6581-6719

Xiao Yi Wang https://orcid.org/0000-0001-8136-6642

Toby R. Petrice https://orcid.org/0000-0003-3764-577X

Therese M. Poland https://orcid.org/0000-0001-7684-1306

### Data availability

All of the data that support the findings of this study are available in the main text.
